# Detection of Tumor DNA in Human Plasma with a Functional PLL-Based Surface Layer and Plasmonic Biosensing

**DOI:** 10.1021/acssensors.1c00360

**Published:** 2021-05-25

**Authors:** Noemi Bellassai, Roberta D’Agata, Almudena Marti, Andrea Rozzi, Stefano Volpi, Matteo Allegretti, Roberto Corradini, Patrizio Giacomini, Jurriaan Huskens, Giuseppe Spoto

**Affiliations:** † Department of Chemical Sciences, University of Catania, Viale Andrea Doria 6, 95122 Catania, Italy; ‡ INBB, Istituto Nazionale di Biostrutture e Biosistemi, Viale delle Medaglie d’Oro, 305, 00136 Roma, Italy; § Department of Molecules & Materials, MESA+ Institute for Nanotechnology, Faculty of Science & Technology, University of Twente, P.O. Box 217, 7500 AE Enschede, The Netherlands; ∥ Department of Chemistry, Life Sciences and Environmental Sustainability, University of Parma, Parco Area Delle Scienze, 17/A, 43124 Parma, Italy; ⊥ Oncogenomics and Epigenetics, IRCCS Regina Elena National Cancer Institute, Via Elio Chianesi, 53, 00144 Rome, Italy

**Keywords:** poly-l-lysine, surface plasmon resonance, cancer diagnosis, plasmonics, peptide nucleic acids

## Abstract

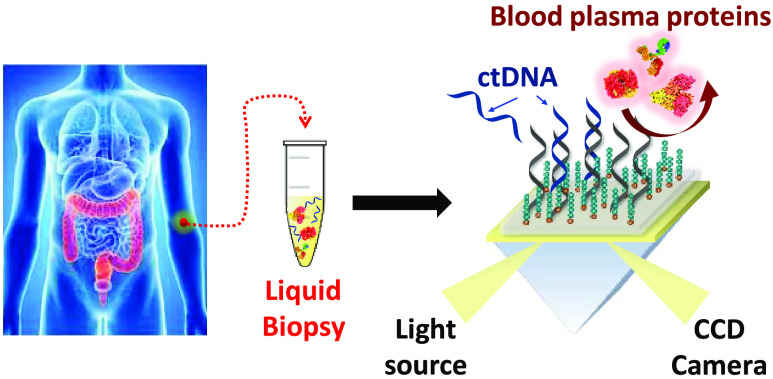

Standard protocols for the analysis of circulating tumor DNA (ctDNA) include the isolation of DNA from the patient’s plasma and its amplification and analysis in buffered solutions. The application of such protocols is hampered by several factors, including the complexity and time-constrained preanalytical procedures, risks for sample contamination, extended analysis time, and assay costs. A recently introduced nanoparticle-enhanced surface plasmon resonance imaging-based assay has been shown to simplify procedures for the direct detection of tumor DNA in the patient’s plasma, greatly simplifying the cumbersome preanalytical phase. To further simplify the protocol, a new dual-functional low-fouling poly-l-lysine (PLL)-based surface layer has been introduced that is described herein. The new PLL-based layer includes a densely immobilized CEEEEE oligopeptide to create a charge-balanced system preventing the nonspecific adsorption of plasma components on the sensor surface. The layer also comprises sparsely attached peptide nucleic acid probes complementary to the sequence of circulating DNA, e.g., the analyte that has to be captured in the plasma from cancer patients. We thoroughly investigated the contribution of each component of the dual-functional polymer to the antifouling properties of the surface layer. The low-fouling property of the new surface layer allowed us to detect wild-type and KRAS p.G12D-mutated DNA in human plasma at the attomolar level (∼2.5 aM) and KRAS p.G13D-mutated tumor DNA in liquid biopsy from a cancer patient with almost no preanalytical treatment of the patient’s plasma, no need to isolate DNA from plasma, and without PCR amplification of the target sequence.

Precision oncology aims at developing treatments that target personalized tumor drivers, e.g., genomic alterations that usually differ from patient to patient. Since tumors evolve, both spontaneously and under therapeutic pressure, cancer drivers change in space and time, making it mandatory to profile cancer through disease stages and therapeutic settings longitudinally.^1^ This has been done for decades with the carcinoembryonic antigen (CEA) and other conventional biomarkers, but liquid biopsy (LB) holds great promise for the straightforward implementation of precision oncology in cancer treatment.^2^ LB provides a minimally invasive approach to detecting circulating tumor cells, circulating tumor DNA (ctDNA), exosomes, and other actionable biomarkers that freely circulate in body fluids of cancer patients, including blood, urine, saliva, and cerebrospinal fluid.^3,4^ In particular, the clinical management of cancer patients has been shown to benefit from the profiling of ctDNA and cell-free DNA (cfDNA) available in the plasma obtained from patients.^5^ Standard protocols for the analysis of ctDNA include the isolation of DNA from the patient’s plasma and its sequence-specific amplification and analysis in buffered solutions.^6,7^ The application of such protocols is hampered by several factors, including the complex preanalytical procedures, the need to process or otherwise stabilize self-decomposing analytes immediately, risks for sample contamination (which increases proportionally to the number of manipulations required), extended analysis time, and consequently assay costs.^8^ Digital bioassays^9^ such as digital PCR (dPCR) have been used to achieve the ultrasensitive detection required to quantify ctDNA in plasma samples with a calibration-free approach and, for this reason, represent the gold standard in current technologies for ctDNA detection in liquid biopsy. However, the obligate preanalytical steps, including cfDNA isolation, and the time-consuming analytical process (2–4 h) represent important limitations for the large-scale implementation of similar bioassays for liquid biopsy analysis.

In contrast, biosensors provide a different approach that may bypass several bottlenecks of current molecular diagnoses. Electrochemical biosensing platforms combined with nanostructured materials have been recently shown to hold promise for simple and direct detection of tumor DNA in the serum^10^ or plasma samples.^11^

When circulating cancer biomarkers contained in a complex biological matrix such as human plasma are revealed directly (i.e., without isolation of the biomarker from the blood before the analysis), the nonspecific adsorption of plasma proteins on the active surface of the sensor may negatively interfere with the biosensing performance by preventing the detection of target biomolecules at low concentrations.^12^ Therefore, while promising, biosensors need improvement. Particularly, their surface architecture requires advanced functionalization, and these should ideally introduce at the same time into the surface layer suitable molecular probes for analyte capture and enhanced antifouling properties. A design combining these two features may be regarded as ideal because it helps minimize nonspecific adsorption of components from the biofluids while maintaining the analytical performances of the biosensing platforms.^13,14^

A recent nanoparticle-enhanced surface plasmon resonance imaging (SPRI)-based assay introduced significant improvements to the direct detection of tumor DNA in the patient’s plasma.^15^ The assay is based on a sandwich detection approach. The volume of plasma sample used for the analysis is a factor of five times smaller than that used by next-generation sequencing (NGS) and dPCR, offering the possibility to multiplex for many distinct DNA sequences and frequently apply the assay in a molecular diagnosis context, with limited amounts of blood, and in rapidly progressing or fragile cancer patients.

Even though the new nanoparticle-enhanced SPRI assay drastically reduces assay time, streamlines diagnostic workflows, and minimizes the potential risk of sample contamination compared to standard methods for liquid biopsy analysis, it still takes 1.5 h to digest plasma proteins in the preanalytical step and specific additional treatments of the SPRI sensor surface are required to minimize the impact of fouling due to plasma components on the assay performances.

Here, we show that the use of a newly designed poly-l-lysine (PLL)-based dual-functional surface layer, combining fouling resistance in the presence of immobilized peptide nucleic acid (PNA) probes, further improves the performance of the nanoparticle-enhanced SPRI assay, allowing us to implement a novel, highly improved, and further simplified protocol for the direct detection of tumor-related DNA in plasma samples. In particular, the new protocol allows direct detection of ctDNA in the plasma after simple (four-step workflow) and rapid (10 min after the initial centrifugation step) preanalytical processing, thus obviating cumbersome and time-consuming preanalytical procedures to isolate and concentrate template DNA and to PCR-amplify target sequences.^16,17^ Issues in the preanalytical step and workflow complexity constitute essential barriers for the clinical adoption of liquid biopsy assays,^18^ and the new method introduces significant improvements in this respect. It minimizes the risk of sample contamination and significantly simplifies the workflow for preanalytical processing compared to the state-of-the-art assays taking 115 min with a multistep workflow involving procedures similar to those included in our four-step workflow.^19,20^ The dPCR and NGS state-of the-art assays dealing with PCR amplification require additional time (between 4 and 18 h) and additional steps in the workflow.^21−25^

The biosensor antifouling coating here described does not include blocking additives, such as surfactants, protein, and nonprotein-based reagents, leading to a reduction in the biorecognition activity of the immobilized probes.^26^

Antifouling surface layers can be obtained with various materials,^27,28^ such as poly(ethylene glycol) (PEG),^29^ single amino acids and peptides,^30^ zwitterionic compounds,^31^ mixed-charge polymers,^32^ and hydrogels.^33^ Among these compounds, PLL, a homopolymer based on repeat units of the cationic l-lysine amino acid, holds remarkable properties as an antifouling material, including superior biocompatibility, predominant hydrophilicity, and good biodegradability.^34^ At physiological pH, the cationic PLL can be deposited on negatively charged surfaces^35,36^ and allows designing monolayers with different functionalities by ensuring critical control over the biosensing interface features.^37,38^ PLL can be modified with various functional groups by introducing neutral or charged side chains,^39−41^ thus offering the possibility to fabricate multifunctional polymeric structures. The main drawback of multifunctional polymers integrating antifouling materials with recognition elements for target capturing is the limited number of target molecules that can access highly packed brush polymers, thereby hampering the biosensing response.^42^ On the other hand, grafting of the recognition elements onto the antifouling layer may negatively affect the antifouling properties of the final layer.^43,44^

The newly designed dual-functional surface layer is based on a mixed-charge PLL polymer structure with antifouling properties.^32^ It comprises an anionic oligopeptide (CEEEEE) and neutral PNA probe side chains. The immobilized oligopeptides, along with the cationic PLL-based polymer, create an antifouling mixed-charge layer, whereas the attached PNA probes provide the target binding partners for the detection of tumor DNA biomarkers directly in human plasma.

The present study aims to thoroughly investigate with SPRI and human plasma the contribution of each component of the dual-functional polymer to the surface layer’s antifouling properties. We also focused on a nanoparticle-enhanced SPRI assay with a dual-functional surface layer containing modified PLL and capture probes based on peptide nucleic acids (PNAs) discriminating wild-type (WT) and Kirsten rat viral sarcoma (KRAS) p.G12D- and p.G13D-mutated genomic DNA in human plasma. The KRAS oncogene is recurrently mutated in different human cancers,^45^ and its mutational status is associated with resistance to treatment with antibodies to EGFR. KRAS testing is now mandatory for the standard of care of colorectal cancers (CRCs), e.g., speeding up and facilitating this diagnostic activity would be valuable for biotechnologists, molecular pathologists, medical oncologists, public decisors, and, obviously, patients. Minimal preanalytical sample treatments and SPRI sensor surface conditioning protocols were required for the analysis. Moreover, the WT and p.G12D-mutated genomic DNA in plasma were detected at the 5 pg μL^–1^ (∼2.5 aM) level together with p.G13D-mutated ctDNA in a liquid biopsy from a CRC patient, specifically with no previous DNA isolation from plasma and PCR amplification.

## Experimental Section

### Chemicals

Reagents were obtained from commercial suppliers and used without further purification. Phosphate-buffered saline tablets (PBS, pH 7.4), poly-l-lysine·HBr (PLL·HBr) (15–30 kDa), Fmoc-Glu(OtBu)-Wang resin, trifluoroacetic acid (TFA), triisopropylsilane (TIS), diethyl ether, hydroxybenzotriazole hydrate (HOBt), 2-(1*H*-benzotriazole-1-yl)-1,1,3,3-tetramethyluronium hexafluorophosphate (HBTU), *N*,*N*-diisopropylethylamine (DIPEA), *N*-methyl-2-pyrrolidone (NMP), piperidine, dichloromethane (DCM), and methanol were purchased from Sigma-Aldrich (The Netherlands). Fmoc-protected PNA monomers and the SPDP-(PEG)_4_ spacer were purchased from LGC-Link (U.K.), and resin for PNA synthesis (Rink Amide ChemMatrix) was purchased from Sigma-Aldrich (Italy). Trisodium citrate dihydrate, tetrachloroauric(III) acid, ethanol, dimethyl sulfoxide, and sodium hydroxide solutions (10 M in water) were purchased from Sigma-Aldrich (Italy). (NHS)-Tetra(ethylene glycol)-maleimide (NHS-(EG)_4_-mal) and Zeba Spin Desalting Columns (7 kD MWCO, 5 mL) were purchased from Thermo Fischer Scientific (The Netherlands). Tris(2-carboxyethyl)phosphine (TCEP) disulfide reducing gel was purchased from Thermo Fischer Scientific (Italy). The oligopeptide CEEEEE (>95% purity grade; MW: 766.73 g mol^–1^) was obtained by Selleck Chemicals LLC (Europe). Pooled human plasma from healthy donors (SER-PLE human recovered plasma frozen; anticoagulant: EDTA 200 mL) was purchased from ZenBio (North Carolina). Phosphate-buffered saline (PBS) solutions at pH 7.4 (137 mM NaCl, 2.7 mM KCl, phosphate buffer 10 mM) were obtained from VWR (Italy). Wild-type streptavidin (WT-SA) was obtained from Invitrogen (Italy). Nitrocellulose membrane filters were purchased from Whatman (U.K.). Gold chips were purchased from Xantec bioanalytics GmbH (Germany). N-α-Fmoc-l-glutamic acid γ-t.-butyl ester (Fmoc-Glu(OtBu)–OH) was purchased from Merk Millipore (The Netherlands). Multisyntech GmbH instrument was employed for the automated solid-phase peptide synthesis (SPPS) of the oligopeptide. Ultrapure water (Milli-Q Element, Millipore) was used for all experiments.

### PNA Probes

We designed PNA probes for wild-type, p.G12D, and p.G13D KRAS sequences to obtain high melting temperatures (higher than 65 °C at 4 μM concentration), as calculated according to an empirical model^46^ with full match complementary DNA sequence. Melting temperatures were estimated for 4 μM concentration using the online available PNA design tool from PNA BIO (http://pnabio.com/support/PNA_Tool.htm) along with Giesen et al.^46^Table 1 shows sequences of the designed and synthesized PNA probes and their acronym.

**Table 1:** Sequences and Acronyms of PNA Probes Used for SPR Experiments^a^

gene, exon	mutational status	PNA probe sequence	acronym
KRAS exon 2	wild-type	SPDP-(PEG)_4_-CTACGCCACCAGCT-Gly-NH_2_	PNA-WT
	p.G12D	SPDP-(PEG)_4_-CTACGCCATCAGCT-Gly-NH_2_	PNA-G12D
	p.G13D	SPDP-(PEG)4- CTACGTCACCAGCT-Gly-NH2	PNA-G13D

a SPDP, *N*-succinimidyl 3-(2-pyridyldithio)propionate; PEG, poly(ethylene glycol).

We synthesized PNA probes by automatic solid-phase synthesis and purified and characterized them as described elsewhere^47^ and briefly reported in the Supporting Information (Figures S2–S5). Protected thiol moieties were introduced in PNA-WT and PNA-G12D probes using commercially available SPDP-(PEG)4-acid formed of 3-(2-pyridyldithio)propionate (SPDP) and four ethylene glycol units that operated as spacers for avoiding steric hindrance to the access to PNA probes (PNA-WT MW: 4219 g mol^–1^; PNA-G12D MW: 4233 g mol^–1^; PNA-G13D MW: 4233 g mol^–1^). We deprotected thiol groups in PNA-WT and PNA-G12D before their use through TCEP disulfide reducing gel. With this aim, 40 μL of TCEP disulfide reducing gel was centrifuged (3300 rpm, 1 min, 25 °C) and the supernatant was removed and discarded. The procedure was repeated twice and then the gel was washed by adding 20 μL of PBS buffer, briefly vortexed to resuspend the gel, and centrifuged (3300 rpm, 1 min, 25 °C). The supernatant was removed and discarded. Then, 20 μL of PNA stock solution was added in two tubes containing the treated gel, vortexed, and incubated for 15 min at 25 °C. After the centrifugation at 25 °C for 1 min, the supernatant containing the thiol-PNA probe was collected. The final concentration of the thiol-PNA solution was determined by UV–vis spectroscopy (Figure S6 and Table S1).

### Synthesis and Functionalization of Gold Nanoparticles

All glassware we used to synthesize and store AuNPs were cleaned with aqua regia (3:1 HCl/HNO_3_) and then with freshly prepared piranha solution (1:3 mixture of 30% H_2_O_2_ and 96% H_2_SO_4_) and thoroughly rinsed with ultrapure H_2_O. We synthesized AuNPs by citrate reduction of HAuCl_4_·3H_2_O according to the method elsewhere described.^48^ Briefly, 20 mL of trisodium citrate (38.8 mM) was quickly added with vigorous stirring to 200 mL of a boiling solution of HAuCl_4_·3H_2_O (1 mM). The color of the solution changed from pale yellow to deep red in a few seconds. A complete reduction of trisodium citrate was obtained after 6–8 min upon boiling. The solution was cooled to room temperature and filtered through a 0.45 μm mixed-cellulose ester membrane filter. Colloidal gold dispersions were stored in the dark and refrigerated at 4 °C. Similar conditions assured nanoparticle stability for several months.

The AuNP dispersion was passivated with poly(vinylpyrrolidone) (PVP) as previously reported^15^ and modified with streptavidin (SA) and then with a biotinylated oligonucleotide sequence (DNA sequence: 5′-CAAGTTTATATTCAGTCAT-3′). Briefly, 500 μL of citrate-stabilized AuNPs (5 nM in water) was added to 500 μL of PVP (50 μM in water). The resulting solution was rotated on a thermomixer for 16–18 h (300 rpm at 23 °C). After the separation of the liquid phase by centrifugation (30 min, 13500 rpm at 23 °C) to pellet the PVP-modified nanoparticles (AuNP@PVP), they were suspended in 50 μL of water. A volume of 25 μL of SA solution (1 mg mL^–1^ in water) was added to 100 μL of AuNP@PVP solution, and the dispersion was mixed for 1.5 h on a thermomixer (300 rpm at 23 °C) to obtain the adsorption of SA on passivated gold nanoparticles (AuNP@PVPSA). After discarding the supernatant by centrifugation (20 min, 13 000 rpm at 23 °C) to pellet particles and separate unbound SA, 2 μL of biotinylated oligonucleotide (100 μM in water) was combined with 20 μL of AuNP@PVPSA diluted to 40 μL with water. The dispersion was mixed and rotated for 2.5 h on a thermomixer (300 rpm at 23 °C). The dispersion was centrifugated for 15 min (13 000 rpm 23 °C) to pellet the particles and discard the supernatant, and AuNP@KRAS were resuspended in 50 μL of water for storage (as the stock solution). The bare and conjugated gold nanoparticles were characterized by UV–vis spectroscopy (VersaWave spectrophotometer, Expedeon Ltd., U.K.), dynamic light scattering, and ζ-potential measurements (Zetasizer Nano ZS ZEN3600, Malvern Instruments, Malvern, U.K.), by diluting the stock solution in PBS buffer (0.1 nM). UV–vis spectra acquired before and after nanoparticles functionalization along with dynamic light scattering and ζ-potential data are shown in Figure S7 and Table S2, respectively.

### Surface Plasmon Resonance Imaging (SPRI)

We performed SPRI experiments using an SPR imager apparatus (GWC Technologies) and analyzed SPR images using V++ (version 4.0, Digital Optics Limited, New Zealand) and Image J 1.32j (National Institutes of Health) software. SPRI pixel intensity values were converted into the percentage of reflectivity (%*R*) using the equation %*R* = 100 × (0.85 *I*_p_/*I*_s_), where *I*_p_ and *I*_s_ refer to p- and s-polarized reflected light, respectively. We obtained SPRI kinetic data by plotting the difference in percent reflectivity (Δ%*R*) from selected regions of interest of SPR images as a function of time. All SPRI experiments were performed at room temperature. We used a microfluidic device with six parallel microchannels (80 μm depth, 1.4 cm length, 400 μm width) to achieve independent control of parallel interactions occurring on the gold chip surface. The device was fabricated using poly(dimethylsiloxane) (PDMS) and connected with PEEK and Tygon tubes (UpChurch Scientific) to a peristaltic pump (IPC, Ismatec SA, Switzerland).

A cleaning procedure of the fluidic system was implemented to minimize the risk of contaminations and memory effects. In particular, after each experiment, the fluidic system was washed with ultrapure water (37 °C, for 2 h) and PBS buffer (at least 1 h). Every 3 weeks, the following cleaning protocol for Tygon tubes was applied: 0.5% sodium dodecyl sulfate (SDS) (10 min), 6 M urea (10 min), 1% acetic acid (10 min), 0.2 M NaHCO_3_ (10 min), and ultrapure water (37 °C, 30 min). Before each experiment, cleaned Tygon tubes were conditioned by flowing PBS buffer at least for 1 h.

### Functionalization of the SPRI Sensor Gold Surface

We cleaned the SPRI gold chip with ultrapure water and ethanol and dried it under N_2_. The chip was then treated with UV-ozone (10 min) and used for SPRI experiments. We synthesized poly-l-lysine-g-maleimide(26%) (PLL-mal(26%)) used for chip functionalization by reaction between PLL and NHS-(EG)_4_-mal, as described elsewhere.^32^ PLL-mal(26%) was adsorbed on the gold chip surface introducing its solution (0.5 mg mL^–1^) in PBS into the microfluidic device in contact with the SPRI sensor surface (flow rate 20 μL min^–1^, 15 min). The surface was then washed with PBS for 10 min to remove unbound PLL-mal(26%). We then freshly deprotected the thiolated PNA probes (0.1 μM in PBS) using TCEP gel and coupled them to the maleimide units of the surface-adsorbed PLL-mal(26%) by thiol-maleimide Michael-type addition to yield PLL-mal(26%)-PNA. We obtained the spatially separated immobilization of the probes by introducing PNA-WT, PNA-G12D, and PNA-G13D solutions (0.1 μM in PBS, flow rate 10 μL min^–1^) into parallel microchannels of the microfluidic device in contact with the PLL-mal(26%)-modified SPRI surface. The dual-functional PLL-mal(26%)-PNA-CEEEEE polymer was obtained by reaction between the thiol moiety in CEEEEE and unreacted maleimide units in PLL-mal(26%)-PNA. With this aim, a CEEEEE solution (1.0 mM in PBS) was adsorbed on PLL-mal(26%)-PNA (flow rate 10 μL min^–1^, 40 min).

### Antifouling Performance of the Modified Surfaces

We evaluated the antifouling capacity of the surface layers by quantifying the surface coverage of protein adsorbed on the functionalized surfaces after exposure to diluted human plasma (10% in PBS) using SPRI. Experiments were conducted using diluted pooled human plasma centrifuged at 10 800 rpm for 10 min at 4 °C and its PBS solutions. First, a stable SPR signal was established by flowing a running buffer (PBS) for at least 5 min (flow rate 10 μL min^–1^). Then, diluted (in PBS) human plasma samples were allowed to flow for 30 min. During the last step of the SPRI experiment, we replaced the human plasma sample with PBS that was flowed for 10 min to wash off weakly adsorbed protein and to establish the final baseline. We tested the antifouling properties of different surfaces modified with the PLL polymer (namely, PLL, PLL with PNA (PLL/PNA), PLL with PNA and CEEEEE (PLL/PNA/CEEEEE; see Table 2, Figures S8 and 4), and PLL-mal(26%) (namely, PLL-mal(26%), PLL-mal(26%) with PNA (PLL-mal(26%)-PNA), and PLL-mal(26%)-PNA-CEEEEE; see Table 2 and Figure 4)). To assess the electrostatic contribution of the peptide to the antifouling activity, CEEEEE and EEEEE were separately adsorbed on the PLL-mal(26%) polymer and the relevant antifouling properties were tested as already described. We synthesized the oligopeptide Glu–Glu–Glu–Glu–Glu (or EEEEE) by the SPPS method using the Fmoc-Glu(OtBu)-Wang resin. EEEEE was purified using high-performance liquid chromatography (HPLC) on a Water (2535) setup, equipped with analytical and preparative XBridge C18 columns, and characterized by mass spectrometry (Figure S1). SPRI kinetic profiles that we detected for the immobilization of the antifouling layers modified with PLL (with and without the maleimide linker) and PLL-mal(26%) with the EEEEE peptide are shown in Figure S9. We quantified the surface coverage of protein adsorbed on the surface of the treated SPRI chips by measuring the variation of Δ%*R* after the injection of the human plasma sample. The mass of adsorbate per unit area (Γ, ng cm^–2^) was estimated based on the theoretical model described by Shumaker-Parry et al.,^49^ where the specific density for the plasma protein was ρ_PP_ = 1.42 g cm^–3^, as the average value of the specific density of plasma proteins,^50^ the refractive index of the plasma protein was *n*_PP_ = 1.53,^51^ and the refractive index of the PBS buffer was 1.33. We also considered the decay length *l*_d_ as 37% of the SPR wavelength.^52^ The sensitivity factor for the SPRI system was *s* = 6009.28%*R*/RIU.

**Table 2:** Surface Coverage (ng cm^–2^) of Plasma Components on PLL-Based Layers^a^

surface layer	notes	Γ (ng cm^–2^)
PLL	no maleimide linker	535 ± 17
PLL/PNA	no maleimide linker	558 ± 54
PLL/PNA/CEEEEE	no maleimide linker	347 ± 45
PLL-mal(26%)	no PNA, no CEEEEE	364 ± 13
PLL-mal(26%)-PNA	no CEEEEE	381 ± 31
PLL-mal(26%)/EEEEE	no PNA, no Cys	213 ± 53
PLL-mal(26%)-CEEEEE	no PNA	48^32^
PLL-mal(26%)-PNA-CEEEEE	dual-functional PLL	46 ± 34

a The standard deviation refers to three independent experiments. Nine replicates were instead considered for PLL-mal(26%)-PNA-CEEEEE.

### DNA Samples and SPRI Detection

Wild-type genomic DNA (gDNA) was isolated from HT-29 human cells, whereas p.G12D-mutated gDNA was isolated from LS174T cells according to the protocol described elsewhere.^53^ Individual plasma samples were obtained from Regina Elena Institutional BioBank. A stage IV colorectal cancer (CRC) patient and a healthy donor donated the anonymized clinical samples we used for the analyses. gDNAs and cfDNAs were KRAS-genotyped by targeted NGS and dPCR according to protocols described elsewhere.^53^ Plasma sample pt#34 (CRC patient) contained 12 215 copies mL^–1^ of p.G13D KRAS-mutated ctDNA and 15 547 copies mL^–1^ of wild-type cfDNA (variant allele frequency 44%). Plasma sample #4 (healthy donor) contained <1 copies mL^–1^ of mutated ctDNA and 1514 copies mL^–1^ of wild-type cfDNA (variant allele frequency < 0.01%).

**Figure 1:**
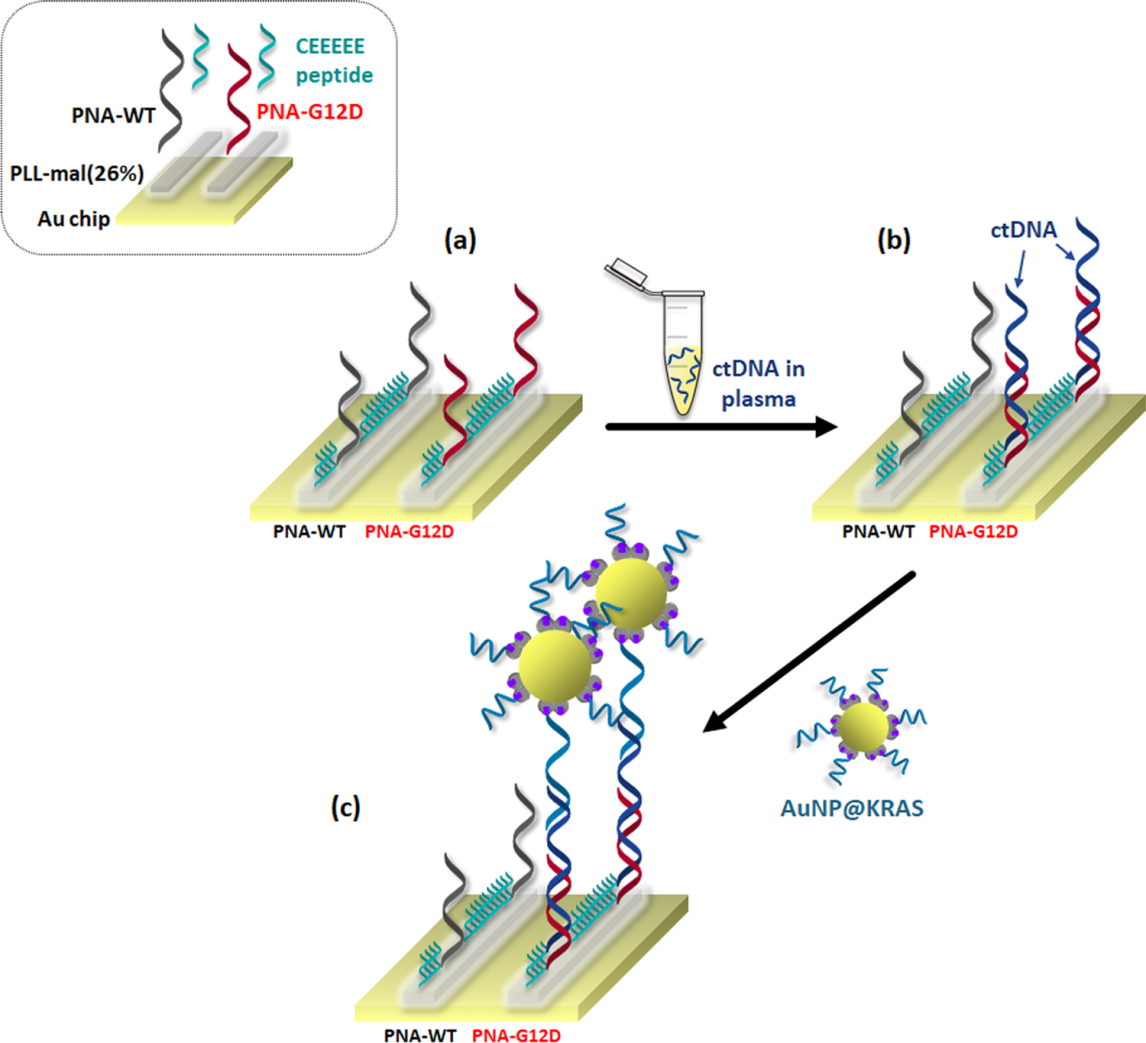
Pictorial representation of the ultrasensitive detection of the p.G12D KRAS-mutated ctDNA sequence in human plasma samples using (a) the dual-functional PLL-based polymer and the nanoparticle-enhanced SPR-based sandwich assay. To simplify the representation, only specifically adsorbed ctDNA is shown. (b) We adsorbed the plasma sample on both PNA-WT and PNA-G12D probes (c) to recognize the mutated DNA sequence and discriminate it from wild-type DNA.

We used the SPRI assay to detect the genetic targets in spiked plasma based on a sandwich detection approach.^54^ PNA probes were covalently bound to PLL-mal(26%) previously adsorbed on the surface of the SPRI gold sensor. CEEEEE was then covalently bound to PLL-mal(26%)-PNA (Figure 1). gDNA in human plasma was then adsorbed on PLL-mal(26%)-PNA-CEEEEE, leading to the hybridization between the relevant PNA probe and the complementary sequence of the target. We used functionalized AuNPs in the last step of the assay to enhance plasmonic detection. With this aim, we decorated AuNPs with an oligonucleotide whose sequence is complementary to a portion of the sequence of the immobilized target not hybridized with the PNA probe. Plasma samples were first centrifuged at 10 800 rpm (4 °C) for 10 min, and then later, the supernatant was diluted in a 1:10 ratio. We detected p.G12D-mutated and wild-type gDNAs spiked in 10% diluted human plasma at a final concentration of 5 pg μL^–1^ (∼2.5 aM).

Before SPRI experiments, we sonicated (3 min, ELMA Transsonic T480/H-2) and vortexed (1 min, IKA Vortex GENIUS 3) spiked plasma samples. gDNAs were denatured by heating (95 °C for 5 min) the spiked plasma sample. Strand reassociation was limited by cooling the treated samples (on ice, 1 min) before their introduction into the SPRI microfluidic device (10 μL min^–1^). We loaded wild-type and mutated gDNA plasma samples into nearby channels of the microfluidic device taking care to swap the order of the loaded samples when moving from an experiment to the following one. The same protocol was adopted for the analysis of liquid biopsy from the CRC patient and individual healthy donor.

## Results and Discussion

### Dual-Functional PLL-mal(26%)-PNA-CEEEEE on a Sensor Surface

**Figure 2:**
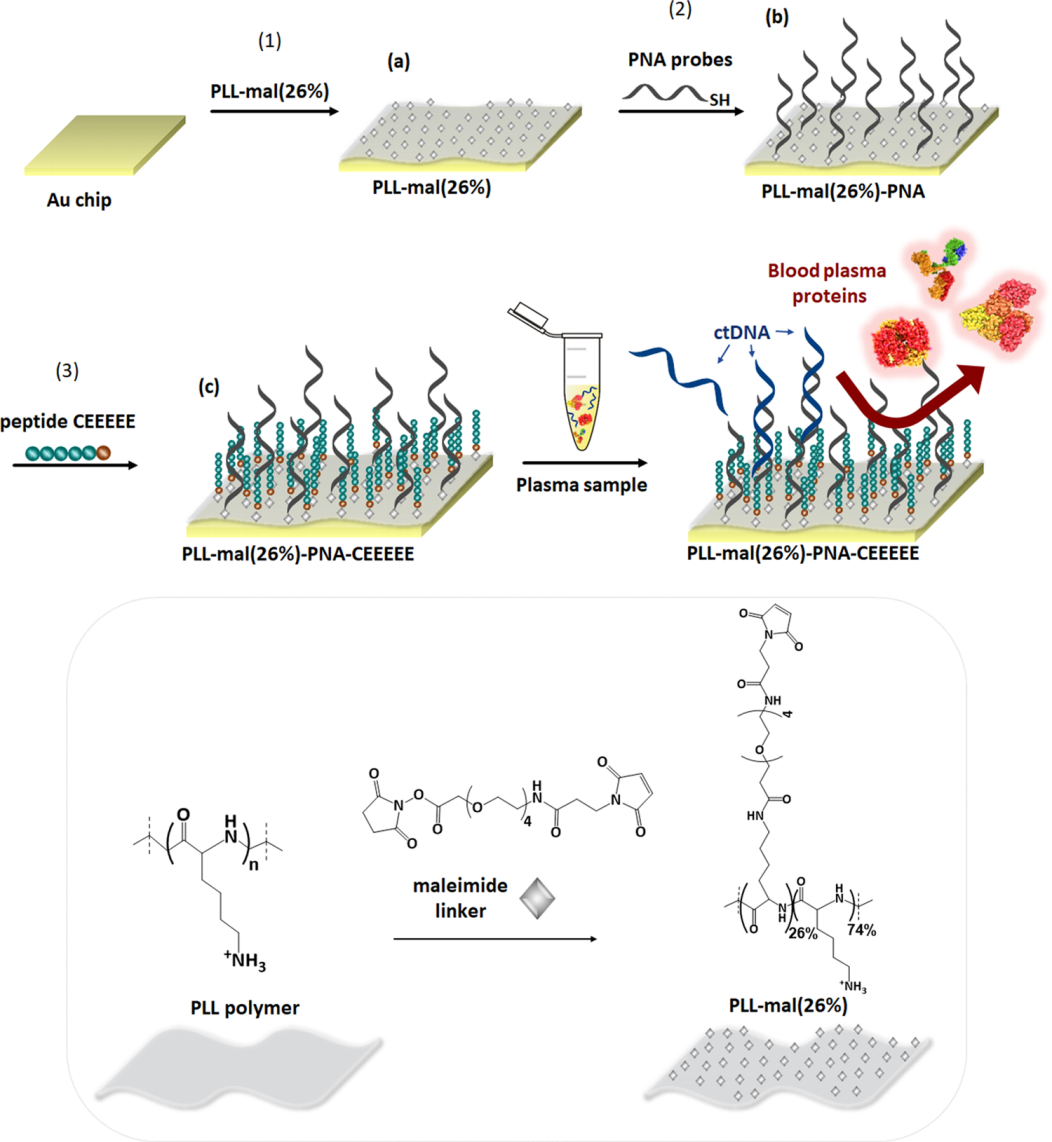
Pictorial representation of (a) PLL-mal(26%), (b) PNA probe, and (c) CEEEEE immobilization on the surface of the SPR gold sensor for the fabrication of the dual-functional PLL-based surface layer. The exposure of the PLL-mal(26%)-PNA-CEEEEE final surface to plasma samples allows both the hybridization of the circulating tumor DNA (ctDNA) target with the complementary PNA probe and the repulsion of plasma proteins.

Figure 2 summarizes the surface functionalization steps we adopted to develop the dual-functional PLL-based polymer used for the direct SPRI detection of cancer biomarkers in human plasma. The PLL-based polymer includes both an anionic oligopeptide (CEEEEE) and neutral PNA probe side chains. The anionic oligopeptides, along with the cationic PLL-based polymer, provide an antifouling mixed-charge layer able to minimize the unspecific adsorption of components in human plasma on the surface. At the same time, PNA probes provide specific recognition of the analyte.

We adsorbed PLL-mal(26%) onto the UV-/ozone-treated surface of an SPRI gold sensor by electrostatic interactions (Figure 2a). For this reason, we introduced a PLL-mal(26%) solution (0.5 mg mL^–1^ in PBS) into the parallel channels of the microfluidic device in contact with the SPRI gold surface. We then coupled thiolated PNA probes (PNA-WT and PNA-G12D, 0.1 μM, 30 min) to the maleimide units of PLL-mal(26%) (Figure 2b). The parallel microchannels of the device provided the separated immobilization of PNA-WT and PNA-G12D probes. The anionic CEEEEE peptide was lastly anchored to residual maleimide moieties of PLL-mal(26%)-PNA (Figure 2c).

We first covalently immobilized PNA probes on PLL-mal(26%) to take under control the probe surface density by monitoring the SPRI kinetic response. The steric hindrance caused by densely immobilized probes may affect the efficiency of the PNA/DNA target hybridization reaction.^55^ In contrast, the immobilized probes’ low surface density limits the number of PNA molecules available for DNA interaction.^56^ A PNA surface coverage of 3 × 10^12^ molecules cm^–2^ has been shown to allow ultrasensitive nanoparticle-enhanced SPRI detection of the DNA target.^15^

**Figure 3:**
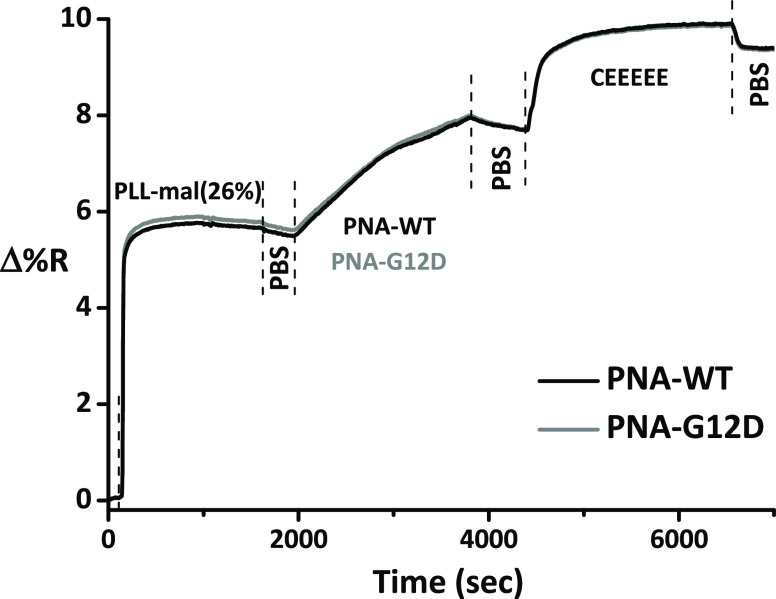
Percent reflectivity (Δ%*R*) over time detected for PLL-mal(26%) deposition, PNA-WT or PNA-G12D immobilization (0.1 μM, 30 min), and CEEEEE (1.0 mM, 40 min) anchoring to the PLL-mal(26%)-PNA layer.

Figure 3 shows representative SPRI kinetic responses detected during the surface functionalization steps mentioned above, leading to the formation of the dual-functional PLL-mal(26%)-PNA-CEEEEE system. After each reactive step (i.e., PLL-mal(26%) adsorption, PNA probe, and CEEEEE immobilization), we washed the surface with PBS to remove unreacted species.

Different kinetic profiles were detected for the immobilization of the three systems composing the functional layer. The PLL-mal(26%) polymer was immobilized on the activated gold surface with fast adsorption kinetics reaching the signal saturation within a few minutes after the electrostatic interaction between the positively charged polymer and the negatively charged gold surface. By contrast, PNA probes and CEEEEE exploit the covalent coupling between thiol groups and maleimide units exposed by the immobilized PLL-mal(26%). Kinetic SPR profiles referring to such interactions indicate a slower reaction than the polymer adsorption on gold, as reported elsewhere.^32^ As expected, the smaller size and higher concentration of CEEEEE compared with PNA probes favors its reaction with maleimide, thereby reaching the signal saturation faster than PNAs. The fabrication of the dual-functional polymer involves consecutive surface functionalization and washing steps with the PBS buffer to remove unreacted species and stabilize the final surface. The last portion of the SPR sensorgram shown in Figure 3 refers to the last PBS washing step. The stable baseline detected during such a step demonstrates the stability of the formed surface layer.

The detected Δ%*R* values, which ultimately depend on the molecular weight of the adsorbed species and on the densities and refractive indexes of both the formed layer and the solution in contact with the surface, allow estimating structural parameters of the adsorbed layer (i.e., thickness and surface coverage) based on the model described by Shumaker-Parry et al.^49^ For the calculation, we considered refractive indexes of PLL-mal *n*_PLL_ = 1.52, PBS buffer *n*_PBS_ = 1.33, CEEEEE *n*_CE_ = 1.44, and PNA probes *n*_PNA_ = 1.40.

The proper immobilization of PNA probes onto the SPR sensor required accurate monitoring of the anchored probes’ surface density to ensure the successful detection of the wild-type or p.G12D-mutated DNA targets. It involves comparing SPRI signals detected from surfaces where the two probes are immobilized. Figure 3 shows the immobilization of PNA-WT and PNA-G12D probes resulting in similar Δ%*R* values corresponding to 5 × 10^12^ molecules cm^–2^. The subsequent immobilization of CEEEEE leads to the formation of a more densely packed layer compared to PNA (21 × 10^12^ molecules cm^–2^). The leveling off of the adsorption curve indicates saturation of sites and/or steric maximization of the surface density. The total surface coverage of PNA and CEEEEE obtained here (26 × 10^12^ molecules cm^–2^) agrees with that estimated based on independent cyclic voltammetry experiments described elsewhere (32 × 10^12^ molecules cm^–2^).^40^

### Contribution of Individual Components to the Antifouling Performance of the Dual-Functional PLL-Based Polymer

To better evaluate the contribution of each functional component of PLL-mal(26%)-PNA-CEEEEE to the antifouling properties of the surface layer, we verified the antifouling behavior of layers, including the functional components with a different combination than in PLL-mal(26%)-PNA-CEEEEE. We tested the antifouling properties of such layers by measuring %*R* changes produced by the running buffer (PBS) after the exposure of the surface to diluted pooled human plasma (10% in PBS, 30 min). Plasma components nonspecifically adsorbed onto the surface are responsible for the detected SPRI signal change. For this reason, we calculated the mass of adsorbate per unit area (Γ) from the measured Δ%*R* value.

**Figure 4:**
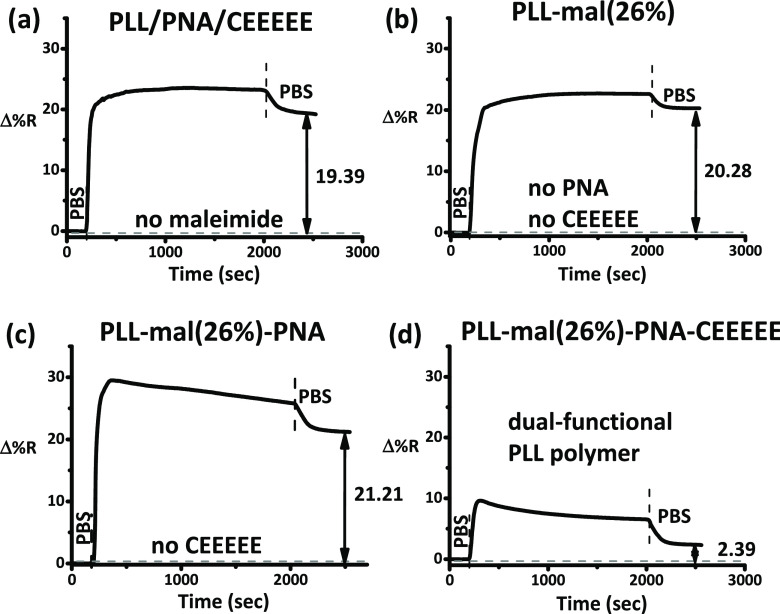
SPRI data for the adsorption of 10% diluted human plasma (30 min) on (a) PLL/PNA/CEEEEE, (b) PLL/PNA, (c) PLL-mal(26%), and (d) PLL-mal(26%)-PNA-CEEEEE layers. PNA-WT was used as the probe.

Figure 4 shows representative SPRI kinetic profiles detected for the exposure of (a) PLL/PNA/CEEEEE (no maleimide groups in PLL), (b) PNA-mal(26%) (no PNA, no CEEEEE), (c) PLL-mal(26%)-PNA (no CEEEEE), and (d) PLL-mal(26%)-PNA-CEEEEE, to 10% plasma in PBS. To test the antifouling polymers, we used the PNA-WT probe. SPRI data for the other investigated layers are reported in the Supporting Information (Figures S8, S10, and S12), whereas SPRI data referring to the surface immobilization of various functional components are reported in Figure S9.

The adsorption of proteins from plasma samples on some of the investigated layers (i.e., PLL-mal(26%)-PNA, PLL-mal(26%)-PNA-CEEEEE, and PLL/PNA; Figures 4b–d and S8) generates an initial SPR signal increase followed by a slowly decreasing kinetic profile. The peculiar profile is the likely consequence of the electrostatic interaction between charged plasma proteins and the charged PLL polymers bearing different amounts of neutral (PNA) and charged (PLL and CEEEEE) species. Similar kinetic profiles have also been reported for the adsorption of plasma proteins on other charged polymers.^57,58^

We tested first PLL-based layers bearing no maleimide functionalities (Figures 4a and S8) to evaluate the role played by maleimide groups in driving the assembly of layers showing enhanced antifouling capacity. Thiol moieties of PNA and CEEEEE structures directly interact with the gold surface of the SPRI sensor (Figure S10). PNA and CEEEEE also may interact nonspecifically with PLL, being the electrostatic interaction between PLL and CEEEEE the primary driving force involved. Such interactions compete with and complement the reaction between thiol moieties and maleimide groups of PLL-mal(26%), causing a nonsignificant change in the number of PNA and CEEEEE molecules adsorbed on sensor surfaces modified with PLL and PLL-mal(26%), respectively. An evident change in the SPRI kinetic profile for CEEEEE adsorption accounting for a transition from a diffusion-limited process to a reaction kinetic-controlled process was observed when maleimide units were present on the surface (Figure S11). The fouling resistance of the surface layers we tested can be assessed through the value of Γ calculated from the SPRI experiments performed with human plasma (Table 2).

All PLL-based layers we tested exhibited some fouling resistance. The adsorption of the neutral PNA probes on cationic PLL produced no significant variation in the fouling resistance of the surface (Γ changed from 535 ± 17 ng cm^–2^ to 558 ± 54 ng cm^–2^). The lowest Γ value (Γ = 347 ± 45 ng cm^–2^) we measured for PLL-based layers bearing no maleimide groups, corresponding to the best fouling resistance, refers to the layer obtained by adsorbing the anionic CEEEEE peptide on PLL/PNA (Table 2 and Figure 4a). Such a result confirmed the critical role of charges on the surface layer’s fouling resistance.

We then tested the fouling resistance of PLL-mal(26%)-based layers bearing maleimide groups. Such layers showed better fouling resistance than the corresponding layers bearing no maleimide units (Table 2). The better fouling resistance of PLL-mal(26%) compared to PLL (Γ = 364 ± 13 vs 535 ± 17 ng cm^–2^) is attributed to the oligo(ethylene glycol) structure of the arm that we covalently added to PLL to introduce maleimide units. As already observed for PLL, the introduction of neutral PNA produced no significant changes in the fouling resistance (Γ = 381 ± 31 ng cm^–2^) of the resulting surface.

The adsorption of CEEEEE on PLL-mal(26%) significantly improved the fouling resistance of the surface as already observed for PLL. In particular, PLL-mal(26%)-CEEEEE provided the best fouling resistance among the tested systems with a remarkable reduction of Γ (48 ng cm^–2^),^32^ which testifies the reduction of the total amount of plasma components adsorbed on PLL-mal(26%)-CEEEEE. The coupling reaction between CEEEEE and the maleimide moiety creates a charge-balanced system that enhances the antifouling capacity of the PLL-mal(26%)-based layer.^32^ At physiological pH, plasma proteins can interact via electrostatic forces with cationic polymers such as PLL and PLL-mal(26%), by raising the mass of adsorbate per unit area (Table 2 and Figure 4a–c). CEEEEE anionic side chains covalently attached to PLL through maleimide groups shield positive charges, thereby reducing the electrostatic attraction of the plasma proteins on the surface (Table 2 and Figure 4d).^32^

The sequential adsorption of PNA and CEEEEE (Figure 3) did not alter the fouling resistance of the PLL-mal(26%)-CEEEEE charge-balanced surface (Γ = 46 ± 34 vs 48 ng cm^–2^^32^), thus offering the opportunity to fabricate a surface layer combining the enhanced fouling resistance with the capacity of PNA probes to hybridize the complementary sequences of DNA in plasma samples specifically.

We tested the fouling resistance of the surface obtained by adsorbing the anionic peptide EEEEE bearing no thiol moiety on PLL-mal(26%) to confirm the synergistic contribution to the antifouling properties of both the covalent coupling between the thiolated CEEEEE and maleimide groups and the repulsive electrostatic interaction between plasma components and the mixed-charge PLL-mal(26%)-based layer. We also detected SPRI curves during the exposure of (a) PLL-mal(26%)/EEEEE and (b) PLL-mal(26%)-CEEEEE surfaces to plasma (10% in PBS) (Figure S12). The two layers provided different Δ%*R* changes both during the adsorption of the diluted human plasma and when the PBS baseline was established after the plasma adsorption. PLL-mal(26%)/EEEEE (Γ = 213 ± 53 ng cm^–2^) did not improve the antifouling property of PLL-mal(26%)-CEEEEE, thus confirming the requirement for the interaction between the thiol group of the anionic peptide and maleimide groups introduced in the PLL structure.

### Nanoparticle-Enhanced SPRI Detection of Tumor DNA in Human Plasma Using the Dual-Functional PLL-Based Polymer

Nanostructure-enhanced SPR has been already shown to allow detection of nucleic acids^15,54,59,60^ including microRNAs,^61^ protein biomarkers,^62,63^ and small molecules^64^ with attomolar sensitivity.^65^ The ultrahigh sensitivity achieved with metallic nanoparticles has been attributed to dielectric constant enhancement from nanoparticles clustered on the SPR chip due to processes such as magnetic interaction^66^ or nucleic acid sequence-induced aggregation.^67^ Possibilities offered by ultrasensitive detection methods based on nanoparticle-enhanced SPR biosensors have been recently reviewed.^68^ Challenges and solutions for ultrasensitive biosensing have also been discussed with a broader perspective.^69^ Here, we demonstrate that the nanoparticle-enhanced SPRI ultrasensitive detection can be implemented for the direct detection of ctDNA in human plasma.

To demonstrate that the PLL-mal(26%)-PNA-CEEEEE surface layer combined with nanoparticle-enhanced SPRI detection of mutated and wild-type DNAs in human plasma provides a new platform for the direct analysis of plasma samples from cancer patients, we adsorbed PLL-mal(26%) on the SPRI gold surface and immobilized PNA probes (0.1 μM in PBS, flow rate 10 μL min^–1^) as already described (Figures 3 and 1a). We obtained the spatial separation of the immobilized PNA probes complementary to wild-type and p.G12D-mutated DNA target sequences using a microfluidic device bearing parallel microchannels (Figure 1). We then introduced CEEEEE (surface density 21 × 10^12^ molecules cm^–2^) to obtain the dual-functional layer.

We analyzed plasma samples (10% in PBS) spiked with p.G12D-mutated or wild-type gDNAs at a final concentration of 5 pg μL^–1^ (∼2.5 aM assuming MW = 1.9 × 10^12^) as a proxy of ctDNA detection in plasma from cancer patients and cfDNA detection in plasma from a healthy donor, respectively.

The spiked samples were introduced in the microfluidic device for adsorption on PNA probes (Figure 1b) after sonication (3 min), vortexing (1 min), heating at 95 °C (5 min), and incubation for 1 min on ice before the analysis.

SPRI responses detected after the adsorption of plasma samples (300 μL, 10 μL min^–1^) spiked with p.G12D-mutated gDNA on PLL-mal(26%)-PNA-CEEEEE layers bearing PNA-WT and PNA-G12D (Figure S13) confirmed the antifouling properties of the surface layer and provided no helpful signal to identify the specific interactions between p.G12D-mutated gDNA and the complementary PNA-G12D probe. After evaluating SPRI responses detected for the adsorption of plasma spiked with wild-type gDNA (Figure S13), we have drawn the same conclusions.

As the last step of the assay, we used functionalized AuNPs to enhance plasmonic detection and produce SPRI signals useful to detect p.G12D-mutated and wild-type gDNAs in plasma samples selectively (Figure 1c). We obtained AuNP@KRAS by conjugating AuNPs with an oligonucleotide complementary to an exposed DNA region of the KRAS exon carrying the p.G12D mutation or the corresponding wild-type sequence not involved in the hybridization with PNA-WT or PNA-G1D probes.^15,70^ We obtained the SPRI signal enhancement by adsorbing AuNP@KRAS dispersion (0.1 nM in PBS. 10 μL min^–1^) on the PNA-functionalized SPRI surface after the already mentioned plasma adsorption and a 10 min washing step with PBS.

**Figure 5:**
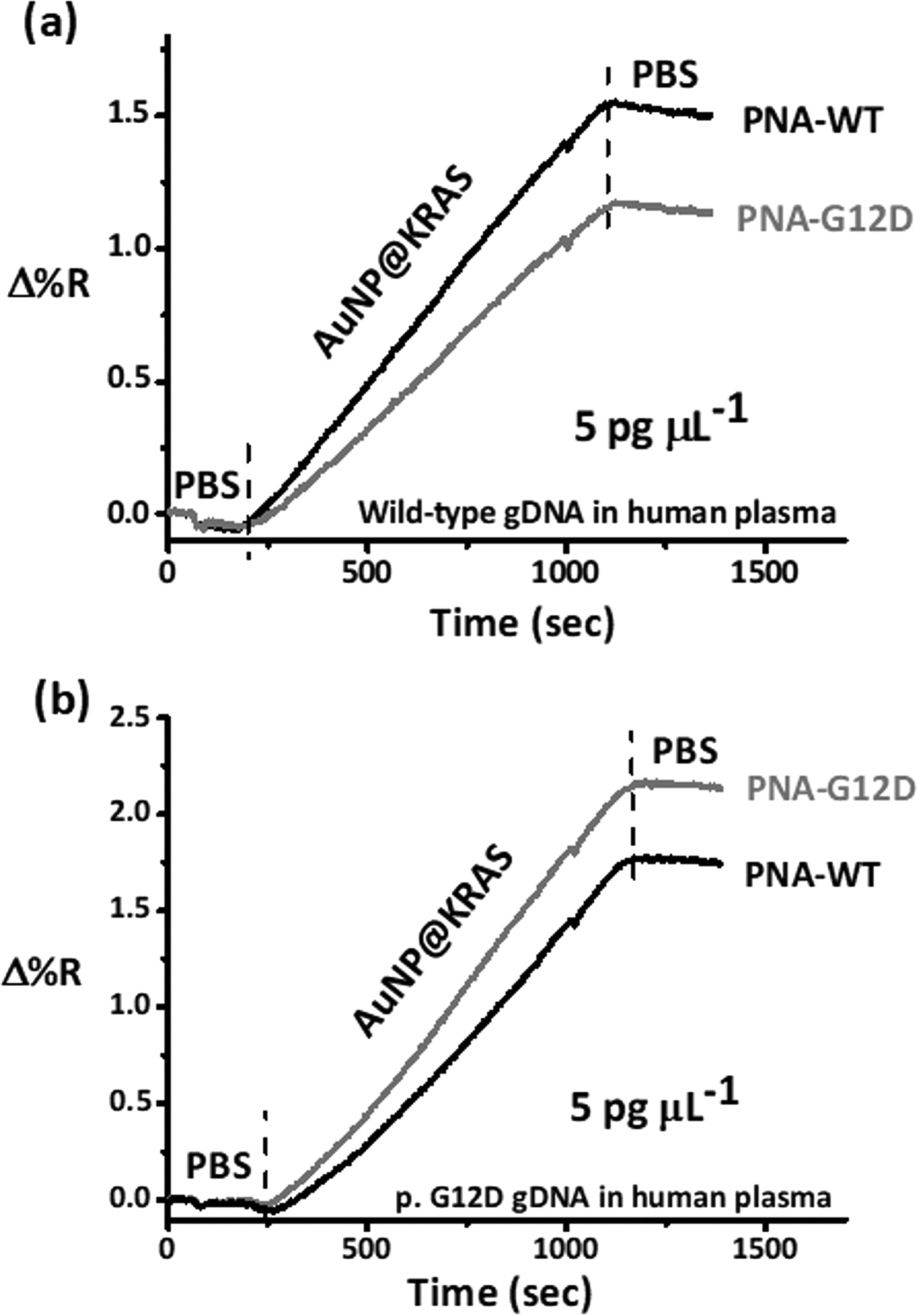
Representative time-dependent SPRI curves for the adsorption of AuNP@KRAS on (a) wild-type and (b) p.G12D-mutated gDNA in plasma previously adsorbed on surface-immobilized PNA-WT and PNA-G12D probes.

After AuNP@KRAS enhancement, the SPRI assay distinguished plasma samples carrying wild-type or p.G12D-mutated gDNAs (5 pg μL^–1^). Nanoparticle enhancement highlighted the preferential interaction of wild-type DNA with PNA-WT (Figure 5a), whereas p.G12D-mutated DNA preferentially interacted with the PNA-G12D probe (Figure 5b), as expected.

In particular, the ratio of Δ%*R* values detected after the adsorption of AuNP@KRAS on surfaces resulting from the interaction of the selected plasma sample with PLL-mal(26%)-PNA-CEEEEE surface layers modified with PNA-WT and PNA-G12D provides better visualization of the discrimination between plasma samples with wild-type or p.G12D-mutated DNA.

The shape of SPR profiles detected for AuNP@KRAS adsorption (Figure 5) is the consequence of diffusion-limited kinetics^71^ arising because the rate of AuNP@KRAS nanoparticle diffusion to the surface is slower than the kinetics of the interaction between oligonucleotides immobilized on AuNP@KRAS and DNA target molecules captured by PNA probes. Figure 5 highlights the difference in SPR signals due to the single base mismatch detection. The detection of WT or p.G12D single base mutated DNAs causes an inversion of the order of curves shown in Figure 5a and 5b, respectively. Nonspecifically adsorbed DNA fragments can trigger nanoparticle aggregation on the surface due to the alteration of the local charge balance they cause, as discussed elsewhere.^67^ Considering the possible contribution of the nonspecific AuNP@KRAS adsorption on the detected SPR signal, we calculated the ratio between SPR signals detected from the two surfaces differing from each other only for the immobilized PNA probe (PNA-WT and PNA-G12D), which differ by only one base. PNA selectivity and cross-reactivity in the PBS buffer have been already tested for similar systems, as reported elsewhere.^15^ The use of PNA clamp sequences and the fine-tuning of the assay temperature may be evaluated to improve the discrimination between AuNP@KRAS enhanced signals.

**Figure 6:**
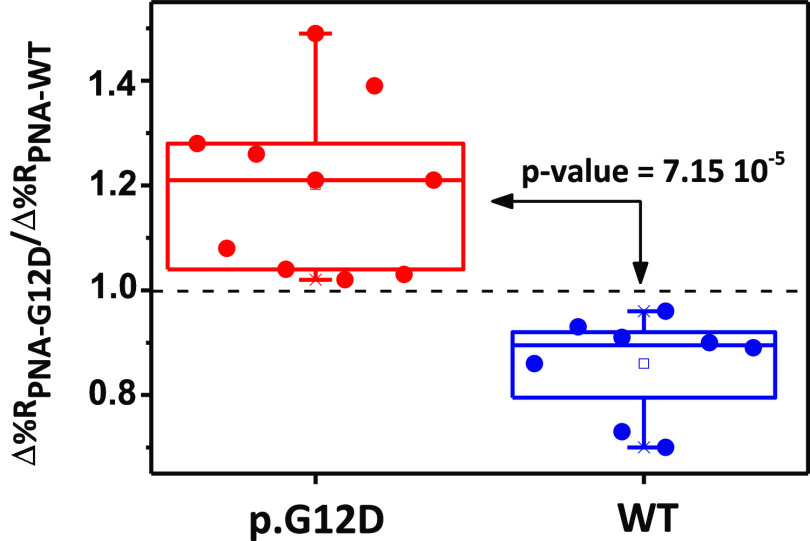
Δ%*R*_PNA-G12D_/Δ%*R*_PNA-WT_ ratio values obtained from replicated experiments aimed at detecting wild-type (WT) and p.G12D gDNAs in 10% diluted human plasma samples (5 pg μL^–1^). Ratios were obtained by considering Δ%*R* values after 1000 s of adsorption of AuNP@KRAS. The ratio considers SPRI responses (Δ%*R*) referred to PNA-G12D (Δ%*R*_PNA-G12D_) and PNA-WT (Δ%*R*_PNA-WT_) probes when the same plasma sample was detected. Wild-type (Δ%*R*_PNA-G12D_/Δ%*R*_PNA-WT_ ratio population mean confidence interval (CI) at the 95% level = 0.86 ± 0.09, replicate measurements *n* = 8) and p.G12D (Δ%*R*_PNA-G12D_/Δ%*R*_PNA-WT_ ratio population mean CI = 1.20 ± 0.16, replicate measurements *n* = 10) samples generated significantly different Δ%*R*_PNA-G12D_/Δ%*R*_PNA-WT_ ratios (*t*-test, level 95%, two-tailed, *p*-value = 7.15 × 10^–5^). A dotted line is shown to highlight better the values of the Δ%*R*_PNA-G12D_/Δ%*R*_PNA-WT_ ratio below and beyond 1.

Figure 6 summarizes results from replicated independent analyses of plasma samples spiked with wild-type or p.G12D-mutated gDNAs (5 pg μL^–1^). We performed the experiments analyzing in parallel wild-type and p.G12D-mutated samples.

The Δ%*R*_PNA-G12D_/Δ%*R*_PNA-WT_ ratio for p.G12D-mutated DNA (population mean confidence interval at the 95% level for the ratio CI = 1.20 ± 0.16, replicate analyses *n* = 10) was significantly different from that for wild-type DNA (Δ%*R*_PNA-G12D_/Δ%*R*_PNA-WT_ 95% CI = 0.86 ± 0.09, *n* = 8; two-tailed *t*-test, level 95%, *p*-value = 7.15 × 10^–5^).

**Figure 7:**
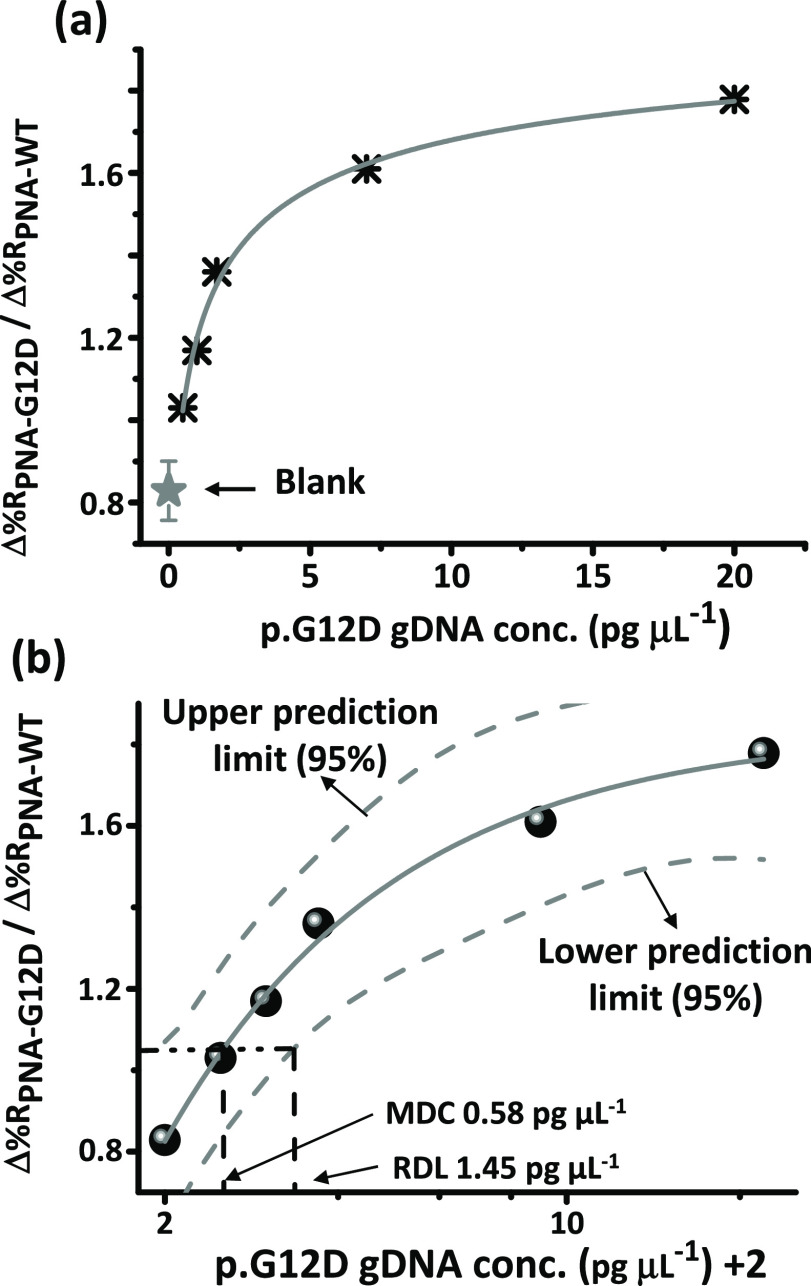
(a) SPRI calibration curve of different p.G12D-mutated gDNA concentrations spiked in 10% diluted plasma of an individual healthy donor (sample #4). The average Δ%*R* ratio generated by blank samples is shown (gray star, *n* = 5). (b) Four-parameter logistic function for experimental data fitting of the SPRI calibration curve is shown (gray line). p.G12D gDNA concentration values are reported on a log-scale axis. We added 2 to the actual concentration to include the negative control (p.G12D concentration = 0) in the fitting procedure, as described in ref (73). The same number was subtracted after the end of the process. The best fit (adj. *R*^2^ = 0.987) was obtained using the equation *y* = *d* + (*a* – *d*)/(1 + ([p.G12D] + 2/*c*)∧*b*) with the following parameters: *a* = −51.3582; *b* = 1.10261; *c* = 0.04994; d = 1.83694. By the data fitting, the minimum detectable concentration (MDC = 0.58 pg μL^–1^) and the reliable detection limit (RDL = 1.45 pg μL^–1^) were estimated as DNA concentrations corresponding to the interpolated intersections of the lower asymptote of the upper 95% prediction limit with the four-parameter logistic fit curve and the lower 95% prediction limit, respectively.

To evaluate the assay performance with a single-donor plasma (sample #4) instead of pooled plasma samples, we generated a calibration curve (Figure 7a) using plasma from an individual healthy donor. In particular, we investigated a dynamic range (0.5–20.0 pg μL^–1^) for p.G12D-mutated DNA in plasma with typical concentrations of ctDNA in liquid biopsy from cancer patients.^72^ The detection of higher ctDNA concentrations involves tuning different parameters, such as nanoparticle concentration and PNA probe surface density, which falls outside of the scope of this paper. We estimated the detection limit of the assay using the four-parameter logistic regression procedure described elsewhere.^73,74^ We used the following four-parameter logistic equation for the experimental data fitting *y* = *d* + (*a* – *d*)/(1 + ([p. G12D] + 2/*c*)∧*b*), where y corresponds to Δ%*R*_PNA-G12D_/Δ%*R*_PNA-WT_ values at the spiked p.G12D concentration [p. G12D] in plasma samples. Figure 7b shows the four-parameter logistic fit (gray line, adj. *R*^2^= 0.990) with lower and upper 95% prediction limits (dashed gray lines). We obtained both the minimum detectable concentration (MDC = 0.58 pg μL^–1^) and the reliable detection limit (RDL = 1.45 pg μL^–1^) as DNA concentrations corresponding to the interpolated intersections of the lower asymptote of the upper 95% prediction limit with the four-parameter logistic fit curve and the lower 95% prediction limit, respectively. The estimated MDC and RDL parameters provide an improved analytical sensitivity compared to other plasmonic platforms recently described for circulating biomarker detection in biofluids.^75^

**Figure 8:**
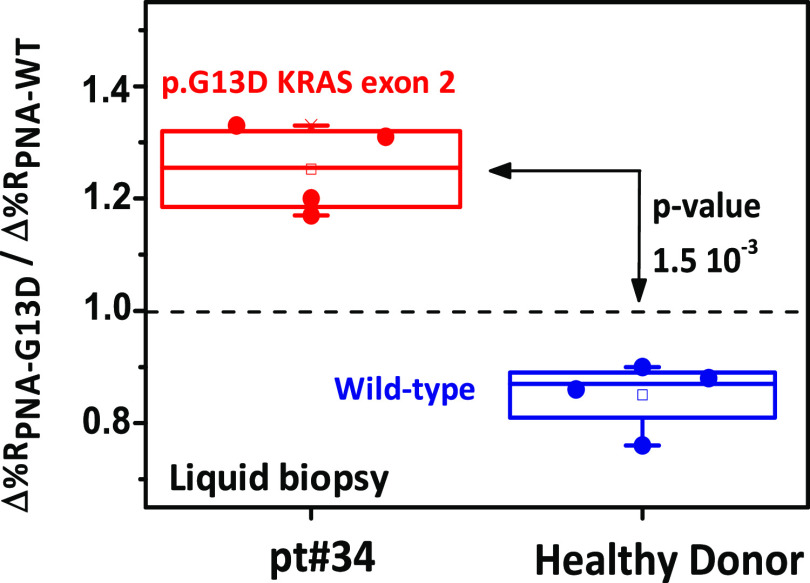
Box plot of the Δ%*R*_PNA-G13D_/Δ%*R*_PNA-WT_ ratio calculated after the adsorption of AuNP@KRAS. Plasma samples from the CRC patient with p.G13D KRAS-mutated ctDNA (sample pt#34) provided values greater than 1, whereas samples from the healthy donor (sample #4, wild-type cfDNA) provided significantly different Δ%*R*_PNA-G13D_/Δ%*R*_PNA-WT_ ratios (*t*-test, level 95%, two-tailed, *p*-value = 1.5 × 10^–3^).

In light of the promising results obtained from experiments with spiked plasma samples, we tested the assay performance for the direct detection of KRAS-mutated ctDNA in plasma of CRC patients as a real-world application of liquid biopsy for cancer diagnosis. With this aim, we analyzed sample pt#34 (CRC patient) with p.G13D KRAS-mutated ctDNA and sample #4 from an individual healthy donor. Figure 8 shows the results (Δ%*R*_PNA-G13D_/Δ%*R*_PNA-WT_) obtained from replicated analyses of liquid biopsies.

The Δ%*R*_PNA-G13D_/Δ%*R*_PNA-WT_ ratio for p.G13D-mutated ctDNA (population mean confidence interval at the 95% level for the ratio CI = 1.25 ± 0.08, replicate analyses *n* = 4) was significantly different from that detected for cfDNA from a healthy donor (Δ%*R*_PNA-G13D_/Δ%*R*_PNA-WT_ 95% CI = 0.85 ± 0.06, *n* = 4; two-tailed *t*-test, level 95%, *p*-value = 1.5 × 10^-–3^).

These data confirm that the designed nanoparticle-enhanced SPRI assay combined with the dual-functional PLL surface layer provides a new platform for the straightforward detection of tumor DNA in plasma samples. The new system introduces significant improvements as compared to the state-of-the-art technologies for liquid biopsy analysis.

The preanalytical workflow of a typical liquid biopsy test envisages DNA isolation from blood and then analysis by either NGS or dPCR. This includes a typical double-spin whole blood/plasma protocol (about 30 min), the purification on an affinity matrix under negative pressure/magnetic field and elution (75 min), and final DNA quantification (5 min).^23^ The subsequent analytical phase lasts from 4 to 18 h (dPCR and NGS, respectively), including the data elaboration time, and this time cannot be compressed because both dPCR and NGS are endpoint methods.^24,25^ In contrast, the new platform significantly cuts down wet-lab time (10 min) and provides a real-time readout. Most importantly, it avoids several cumbersome steps, e.g., temperature cycling, sample carryover, sample transfer to different pieces of equipment such as different types of centrifuges (a wide range of g forces is normally needed), purification devices, fluorimeter, and test tubes for volume/concentration adjustments.^18^ Altogether, the total preanalytical turnaround time is 115 vs 10 min for conventional testing and dual-functional PLL SPRI, respectively. The analytical time is 4–18 vs 2 h. The total turnaround time is 350 min or 48 vs 2.5 h. The hands-on time is rather similar, but the new SPRI assay condensates all steps within a narrow time-lapse, whereas NGS requires at least an overnight step and the person-time intensive use hands-off time is fractionated and poorly usable.

The new platform also introduces substantial improvements compared to the recently introduced nanoparticle-enhanced SPRI assay for detecting ctDNA in plasma samples.^15^ The preanalytical processing of the plasma sample no longer involves the 1.5 h lasting treatment of plasma samples with proteinase K, and the SPRI sensor surface is not treated with dithiothreitol or other blocking additives after the plasma adsorption as a consequence of the surface fouling resistance introduced by PLL-mal(26%)-PNA-CEEEEE.

## Conclusions

This paper demonstrates that the combined use of the new PLL-mal(26%)-PNA-CEEEEE surface layer and nanoparticle-enhanced SPRI provides a platform for the straightforward detection of tumor DNA in the plasma of cancer patients. In particular, the key achievement described herein is the design of a new dual-functional low-fouling PLL-based polymer, containing both anionic oligopeptide and neutral PNA probe side chains. We show that this very focused technical improvement has a considerable implication because it virtually eliminates sample pretreatment from patient serum samples’ processing. In other words, blood may be processed for liquid biopsy exactly as for routine blood biochemistry, virtually eliminating dedicated liquid biopsy pipelines, and contributing to fit liquid biopsy into the standard hematological routine. The densely immobilized CEEEEE peptides and the cationic PLL-based structure create a mixed-charge layer that inhibits the unspecific adsorption of components of complex matrices such as the human plasma. At the same time, the sparsely attached PNA probes provide the genomic target binding partners. We have demonstrated that components of the dual-functional PLL polymer perform a synergistic antifouling effect. We have studied the role played by each component of the antifouling surface layer by providing information that may be useful for the development of different biosensing platforms. Nanoparticle-enhanced SPRI sandwich assays using the new dual-functional surface layer enable the specific detection of tumor KRAS single-point mutated gDNA, at the attomolar level and directly in human plasma, and ctDNA in a liquid biopsy sample from a CRC patient with no need for preliminary ctDNA isolation, purification, and amplification. Thus, the new platform is not subject to several constraints affecting methods currently applied in the clinical practice for cancer diagnosis, which, instead, require both complicated and time-consuming sample processing and PCR amplification of ctDNA. PLL-mal(26%)-PNA-CEEEEE antifouling properties also simplify the nanoparticle-enhanced SPRI assays compared to a recently introduced NESPRI-based approach.

In conclusion, the SPRI biosensing strategy based on PLL-mal(26%)-PNA-CEEEEE offers a rapid, amplification-free, and straightforward detection of tumor-derived materials circulating in biological fluids and makes a significant contribution to the improvement of early clinical diagnosis and personalized medicine in liquid biopsy. Hopefully, biosensors with low-fouling surfaces will contribute a much-needed technical solution and will make liquid biopsy a truly routine assay, widely available, and fully integrated into advanced health-care systems.

## Supporting Information Available

Characterization of oligopeptide; PNA probes and gold nanoparticles; and SPRI data (PDF)
