# Supplementary material for: Detection of Tumor DNA in Human Plasma with a Functional PLL-Based Surface Layer and Plasmonic Biosensing
Source: ACS Sens. 2021 May 25;6(6):2307–19. doi: 10.1021/acssensors.1c00360 (PMC8294610; doi:10.1021/acssensors.1c00360)
Supplement: Supplementary file 1 — se1c00360_si_001.pdf [file se1c00360_si_001.pdf]

Supporting Information

Detection of tumor DNA in human plasma with a functional PLL-based surface layer and plasmonic biosensing

Noemi Bellassai[1], Roberta D'Agata[1,2], Almudena Marti[3], Andrea Rozzi[4], Stefano Volpi[4], Matteo Allegretti[5], Roberto Corradini[2,4], Patrizio Giacomini[5], Jurriaan Huskens[3]*, Giuseppe Spoto[1,2]*

1 Department of Chemical Sciences, University of Catania, Viale Andrea Doria 6, 95122, Catania, Italy.

2 INBB, Istituto Nazionale di Biostrutture e Biosistemi, Viale delle Medaglie d'Oro, 305, 00136 Roma, Italy.

3 Department of Molecules & Materials, MESA+ Institute for Nanotechnology, Faculty of Science & Technology, University of Twente, P.O. Box 217, 7500 AE Enschede, The Netherlands.

4 Department of Chemistry, Life Sciences and Environmental Sustainability, University of Parma, Parco Area Delle Scienze, 17/A, 43124, Parma, Italy.

5 Oncogenomics and Epigenetics, IRCSS Regina Elena National Cancer Institute, Via Elio Chianesi, 53, 00144 Rome, Italy.

* To whom correspondence should be addressed: **j.huskens@utwente.nl, giuseppe.spoto@unict.it**

Table of contents

Figure S1. Mass spectrum of oligopeptide EEEEE after purification, using 90 % Milli-Q water and 10 % acetonitrile.

Figure S2. UPLC/MS chromatogram of synthesized PNA probe for p.G12D mutation with protected thiol group.

Figure S3. Mass spectrum (from 0 to 1100 m/z) of synthesized PNA probe for p.G12D mutation with protected thiol group.

Figure S4. UPLC/MS chromatogram of synthesized PNA probe for p.G13D mutation with protected thiol group.

Figure S5. Mass spectrum (from 0 to 1500 m/z) of synthesized PNA probe for p.G13D mutation with protected thiol group.

Figure S6. Optical absorption spectra of synthesized PNA Wild-type, PNA probes for p.G12D and p.G13D mutations before and after TCEP reduction.

Figure S7. Optical absorption spectra of AuNPs dispersions after the functionalization. Bare AuNPs dispersions exhibit a localized SPR peak at 520 nm that shifted at 528 nm after conjugation with 3'biotinilated KRAS exon 2 oligonucleotide (AuNP@KRAS).

Figure S8. SPRI adsorption curves of plasma proteins on PLL (no maleimide linker, no PNA, no CEEEEE) and PLL/PNA (no maleimide linker, no CEEEEE) after the exposure of diluted human plasma solution.

Figure S9. SPRI responses and kinetic profiles detected for PLL, PLL/PNA and PLL/PNA/CEEEEE (no maleimide groups in the structure); PLL-mal(26%) (no PNA, no CEEEEE) and PLL-mal(26%)-PNA (no CEEEEE); PLL-mal(26%)/EEEEE (no PNA, no Cys).

Figure S10. SPRI responses and kinetic profiles detected for the immobilization of PNA/CEEEEE on gold surface and SPRI adsorption curves of plasma proteins on PNA/CEEEEE.

Figure S11. SPRI responses and kinetic profiles detected for the immobilization of CEEEEE on PLL surface and PLL-mal(26%) surface.

Figure S12. SPRI kinetic data for the adsorption of 10% human plasma on (a) PLL-mal(26%)/EEEEE and (b) PLL-mal(26%)-CEEEEE.

Figure S13. Experimental SPRI curves, in percent reflectivity (Δ%R) over time, of gDNA on PLL-mal(26%)-PNA-CEEEEE layers bearing PNA-WT and PNA-G12D respectively using 5 pg μL^{-1} of ctDNA fragments in diluted human plasma samples.

Table S1. PNA-WT, PNA-G12D and PNA-G13D concentrations and yield of TCEP reduction.

Table S2. Properties of bare (AuNPs) and functionalized gold nanoparticles (AuNP@KRAS) used for ctDNA detection in SPR assay.

Table S3. $\Delta\%R_{PNA\ G12D}$, $\Delta\%R_{PNA\ WT}$ and $\Delta\%R_{PNA\ G12D}/\Delta\%R_{PNA\ WT}$ values obtained from twelve replicated experiments aimed at detecting 5 pg μL^{-1} solutions of WT and G12D non-amplified genomic DNAs in human plasma samples after 1000 s of adsorption of conjugated AuNPs.

Synthesis of oligopeptide EEEEE

The oligopeptide Glu-Glu-Glu-Glu-Glu (or EEEEE) was synthesized by SPPS using the Fmoc-Glu(OtBu)-Wang resin. The linker attached to the polystyrene core is a 4-hydroxybenzyl alcohol moiety, modified with glutamate (OtBu)-OH used as the first amino acid. In every cycle, one glutamic acid was attached to the peptide sequence. The active ester group for the carboxyl groups was introduced as a phosphonium salt of a non-nucleophilic anion (hexafluorophosphate) using the HBTU/HOBt coupling activation and a DIPEA solution (0.52 M) in NMP solvent. Thoroughly, four vials of the peptide were prepared by weighing 50 mg of Fmoc-Glu(OtBu)-Wang resin per vial. Thereafter, the resin was filled with NMP for the swelling phase for 2 h. Fmoc-Glu(OtBu)-OH (0.29 M) was dissolved in HOBt/HBTU (0.30 M/0.26 M) solution in NMP solvent for the coupling activation of glutamic acids. Before the addition of the free glutamic acid, 20% of piperidine in NMP removed the Fmoc group and deprotected the amino acid at the end of peptide sequence. This deprotection step was repeated for five cycles of the synthesis. The peptide was rinsed with NMP, DCM, and methanol (three cycles for three hours); then, the product was dried overnight under vacuum. To remove EEEEE peptide from the resin and deprotect all of the pendant groups, a cocktail cleavage TFA/TIS/Milli-Q H_2O (95%/2.5%/2.5%) was utilized. During the cleavage, the peptide was gently stirred for 4 h to avoid breaking the resin. Subsequently, the resin was rinsed with TFA, and all of the organic scavengers were removed using the rotavapor.

For the precipitation, diethyl ether was added to the peptide, and the sample was collected after three/four centrifugation steps. Afterward, the precipitate was dissolved, lyophilized, and stored under Argon flow to prevent the degradation of the peptide. The purification of EEEEE was performed using high-performance liquid chromatography (HPLC) on Water (2535) setup equipped with analytical and preparative XBridge C18 columns. The peptide solution was dissolved in H_2O with 0.1% TFA and purified by gradient elution method (linear gradient from H_2O (99%) to acetonitrile (ACN, 100%)). The retention time of the oligopeptide was 10 min. The oligopeptide was characterized by mass spectrometry (Fig. S1).

Synthesis of PNA probes

PNA-WT was synthesised and characterized in a previous work.[1] PNA-G12D was synthesized using Fmoc strategy and automatic Syro I peptide synthesizer, using Fmoc-PNA(Bhoc) monomers, on a Rink amide ChemMatrix resin loaded with Fmoc-Gly-OH as first monomer (0.2 mmol/g), 5 equivalents of each monomer and HBTU/DIPEA as activating mixture. All other details were as described in a recent protocol. Linkage of the SPDP moiety was achieved using HBTU/DIPEA overnight coupling. Cleavage of the resin was performed using a TFA/m-cresol 9:1 solution for 1.5 h, twice. The purity and identity of intermediate sequences and of the final PNAs were evaluated by cleavage of a minimal amount of the resin and analysis by UPLC-ESI/MS. The crude PNA probes were cleaved from the resins, purified by HPLC and characterized by UPLC/ESI-MS and UV/Vis techniques. RP-HPLC purifications were performed on an Agilent 1260 Infinity II Series instrument with a Phenomenex Luna C18 column (250 x 4.6 mm, 5 μm) at 40°C. Samples were injected by Agilent 1260 Vialsampler – G7129A unit, while fractions were collected by an Agilent 1260 FC-AS – G1364 C unit. A flow rate of 4 mL/min was used with the following solvent systems: (A): 0.1% TFA in H_2O and (B): 0.1% TFA in MeCN. The column was flushed for 5 min with solvent A, then a gradient from 0 to 50% B in 30 min and to 100% B in 1 min; then final wash for 5 min in 100%, before restoring initial conditions.

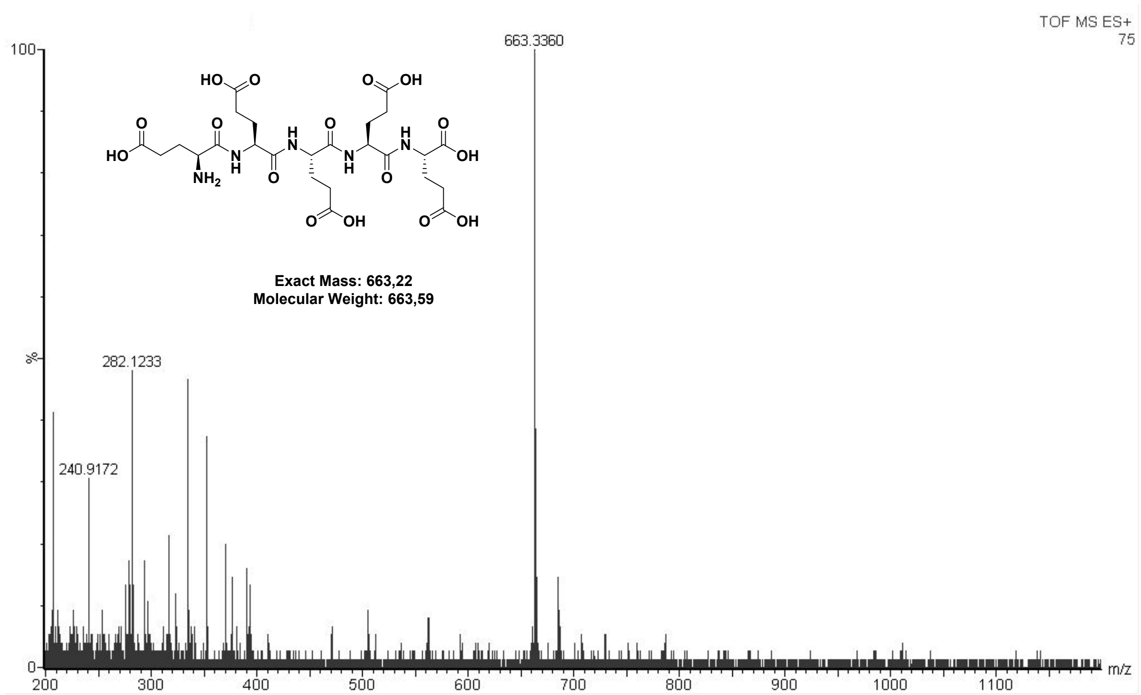

Figure S1. Mass spectrum of oligopeptide EEEEE after purification, using 90 % Milli-Q water and 10 % acetonitrile.

PNA-G12D (AR5-082 G12D): SPDP-(PEG)$_4$- CTACGCCA**T**CAGCT -Gly-NH$_2$

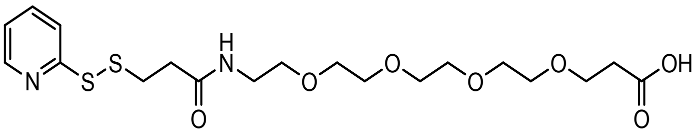

SPDP-(PEG)$_4$ spacer

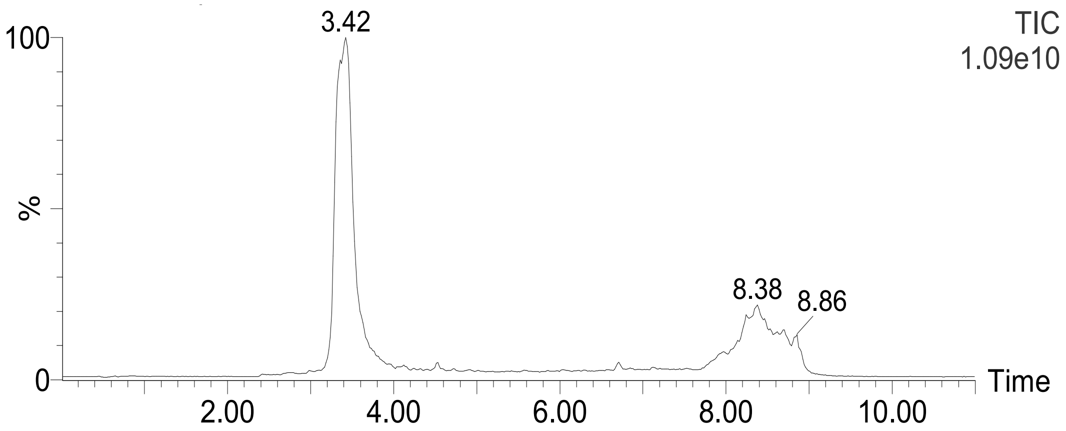

Figure S2. UPLC/MS chromatogram of synthesized PNA probe for p.G12D mutation with protected thiol group. Column: Waters Acquity UPLC BEH C18 column (2.1x50 mm, 1.7 µm), t: 35°C; flow rate of 0.25 mL/min. Eluents (A): 0.2% FA in H_2O and (B): 0.2% FA in MeCN (FA = formic acid). Gradient: isocratic for 0.9 min with solvent A, then gradient to 50% B in 5.7 min.

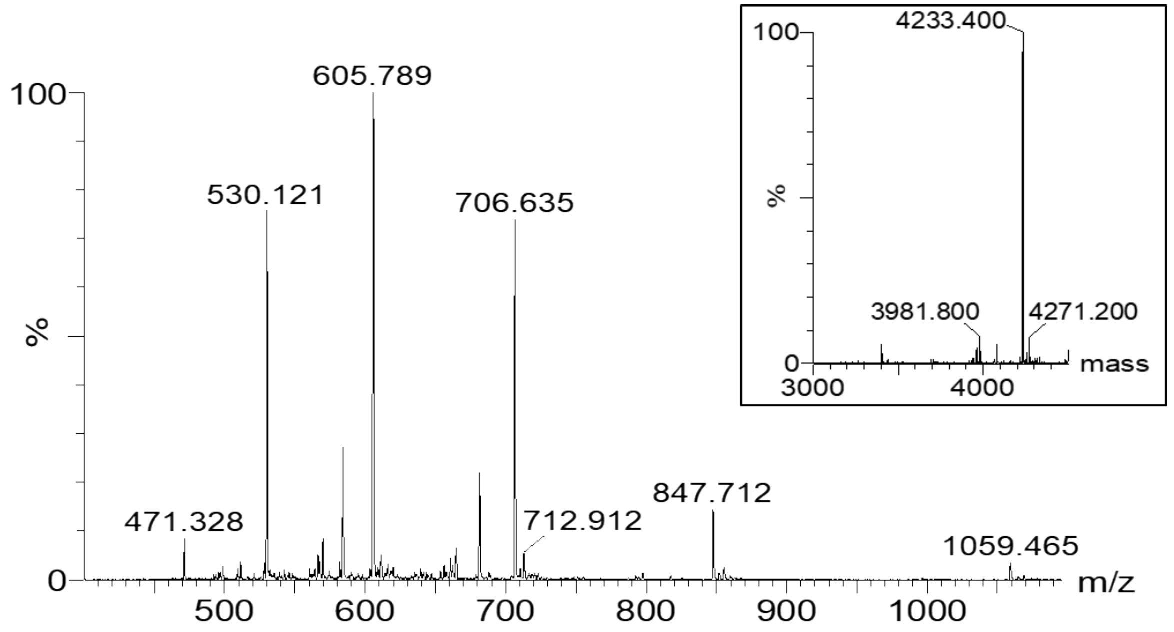

Figure S3. Mass spectrum (from 0 to 1100 m/z) of synthesized PNA probe for p.G12D mutation with protected thiol group *(peak at 3.42 min of the chromatogram in figure S2)*. Multicharge: calculated m/z =1059.57 $[M+4H]^{4+}$, 847.66 $[M+5H]^{5+}$, 706.71 $[M+6H]^{6+}$, 605.76 $[M+7H]^{7+}$, 530.16 $[M+8H]^{8+}$, 471.36 $[M+9H]^{9+}$. Inset: deconvoluted mass from 3000 to 4500 uma; MW: 4233.86 (calc.), 4233.4 (found).

PNA-G13D (SV02-135 G13D): SPDP-$(PEG)_4$-SPDP-CTACG**T**CACCAGCT-Gly-NH_2

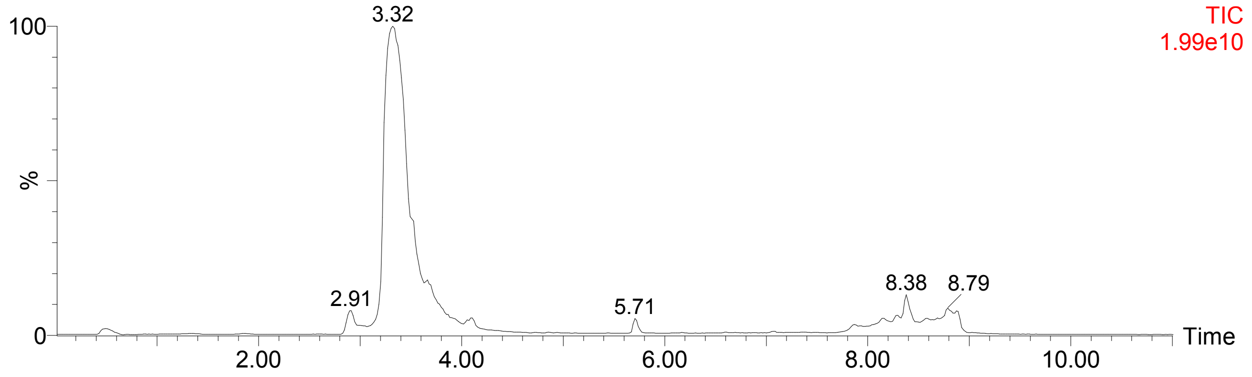

Figure S4. UPLC/MS chromatogram of synthesized PNA probe for p.G13D mutation with protected thiol group. Column: Waters Acquity UPLC BEH C18 column (2.1x50 mm, 1.7 µm), t: 35°C; flow rate of 0.25 mL/min. Eluents (A): 0.2% FA in H_2O and (B): 0.2% FA in MeCN (FA = formic acid). Gradient: isocratic for 0.9 min with solvent A, then gradient to 50% B in 5.7 min.

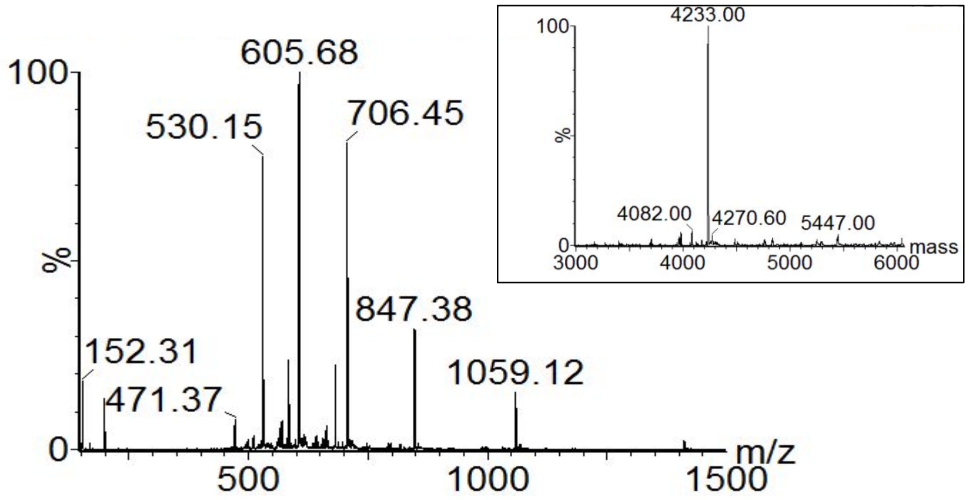

Figure S5. Mass spectrum (from 0 to 1500 m/z) of synthesized PNA probe for p.G13D mutation with protected thiol group *(peak at 3.32 min of the chromatogram in figure S4)*. Multicharge: calculated m/z =1059.12 $[M+4H]^{4+}$, 847.38 $[M+5H]^{5+}$, 706.45 $[M+6H]^{6+}$, 605.68 $[M+7H]^{7+}$, 530.15 $[M+8H]^{8+}$, 471.37 $[M+9H]^{9+}$. Inset: deconvoluted mass from 3000 to 6000 uma*;* MW*:* 4233.3 (calc.), 4233.0 (found).

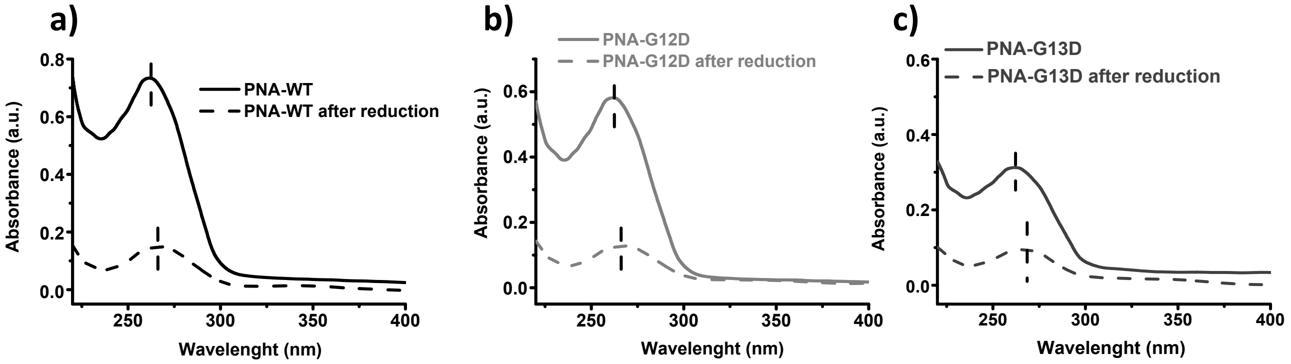

Figure S6. Optical absorption spectra of (a) synthesized PNA Wild type probe with protected thiol group (black solid line) and after TCEP reduction (black dash line); (b) synthesized PNA probe for p.G12D mutation with protected thiol group (grey solid line) and after TCEP reduction (grey dash line); (c) synthesized PNA probe for p.G13D mutation with protected thiol group (dark grey solid line) and after TCEP reduction (dark grey dash line).

Table S1. PNA-WT, PNA-G12D and PNA-G13D concentrations and yield of TCEP reduction. Standard deviation was calculated for six replicates for PNA-WT, PNA-G12D and four replicates for PNA-G13D.

	**Concentration (μmol L^{-1})**	**Yield (%)**
PNA-WT after reduction	15±4	31.2
PNA G12D after reduction	14±2	30.7
PNA G13D after reduction	7±1	25.6

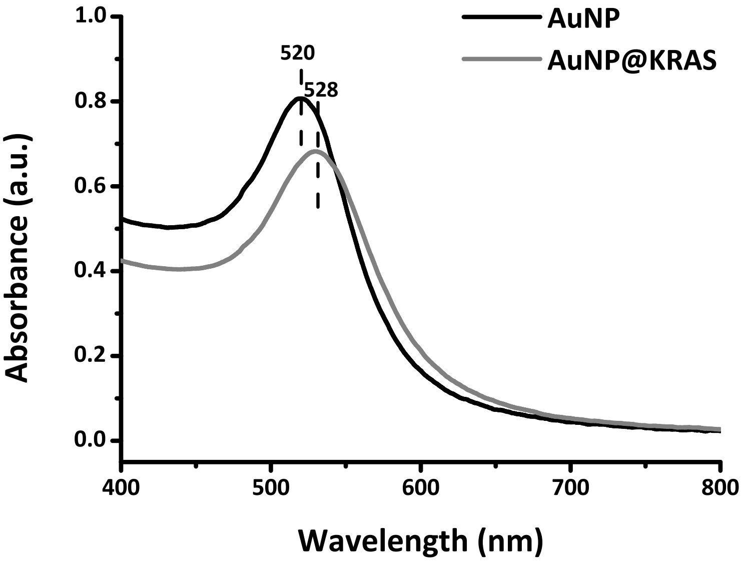

Figure S7. Optical absorption spectra of AuNPs dispersions after the functionalization. Bare AuNPs dispersions exhibit a localized SPR peak at 520 nm that shifted at 528 nm after conjugation with 3'biotinilated KRAS exon 2 oligonucleotide (AuNP@KRAS).

Table S2. Properties of bare (AuNPs) and functionalized gold nanoparticles (AuNP@KRAS) used for ctDNA detection in SPR assay.

	**ξ ± sd (mV)**	**Z-aver ± sd (nm)**	**PDI ± sd**
**AuNPs**	-41 ± 8	21.7 ± 0.7	0.19 ± 0.02
**AuNP@KRAS**	-27 ± 3	51.5 ± 0.7	0.185 ± 0.008

ζ= Zeta potential; Z-aver= Z-average radius; PDI= Polydispersity index from DLS measurements

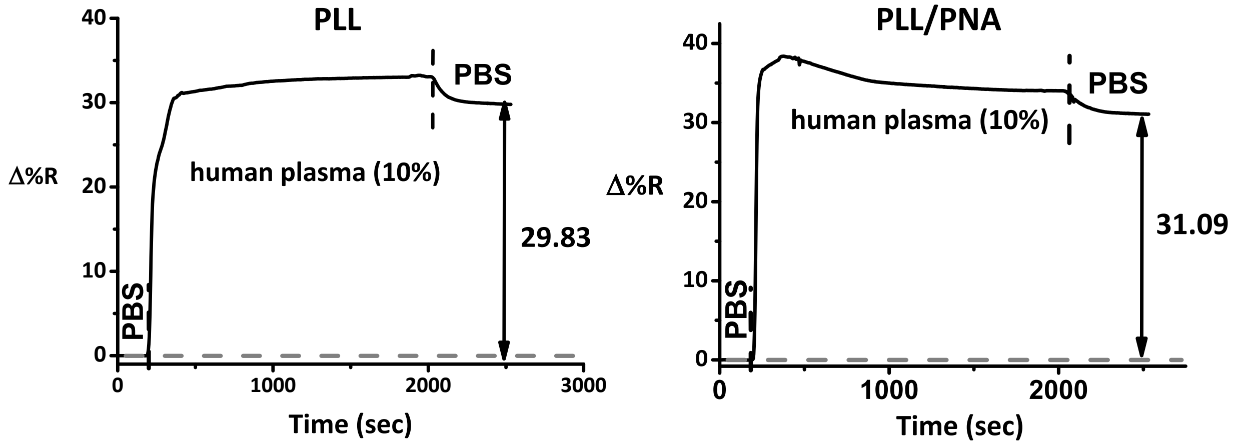

Figure S8. SPRI adsorption curves of plasma proteins on PLL (no maleimide linker, no PNA, no CEEEEE) and PLL/PNA (no maleimide linker, no CEEEEE) after the exposure of diluted human plasma solution (10%) for 30 minutes. PNA-WT sequence was used as PNA probe. The antifouling tests were replicated in three independent experiments.

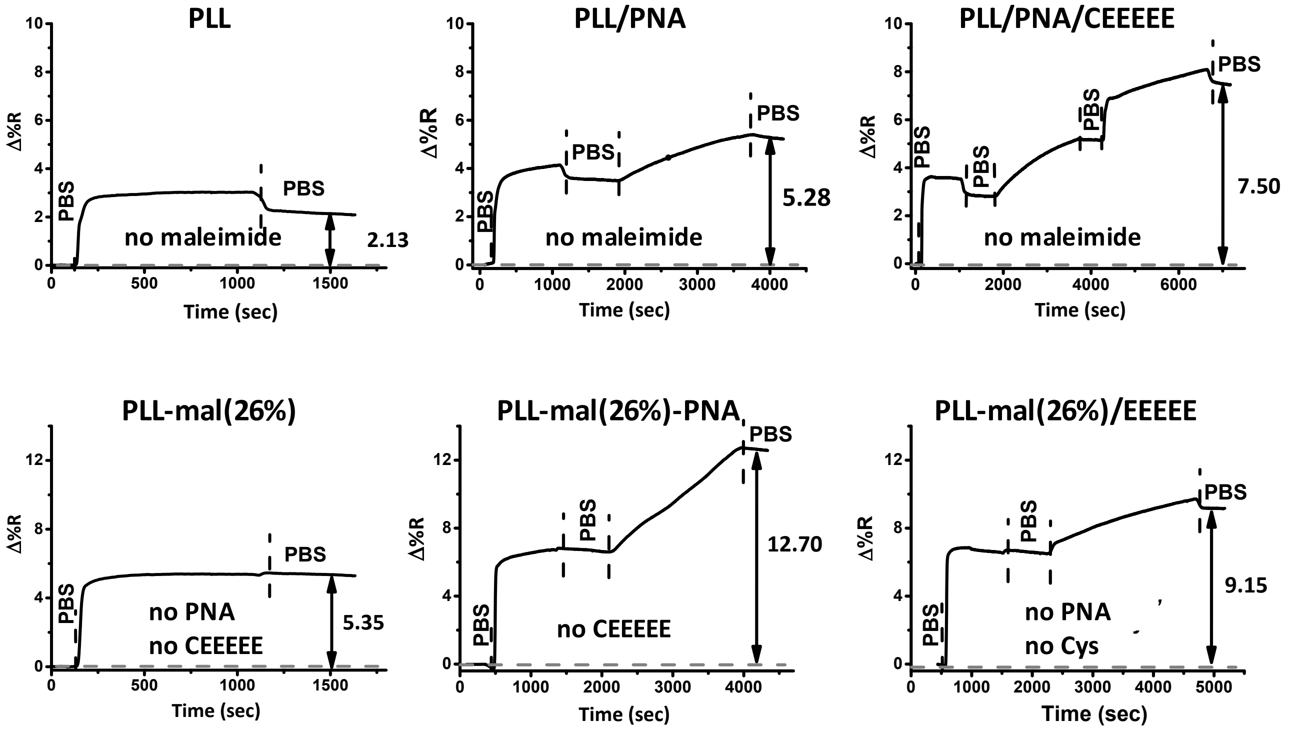

Figure S9. SPRI responses and kinetic profiles detected for PLL, PLL/PNA and PLL/PNA/CEEEEE (no maleimide groups in the structure); PLL-mal(26%) (no PNA, no CEEEEE), PLL-mal(26%)-PNA (no CEEEEE) and PLL-mal(26%)/EEEEE (no PNA, no Cys). PNA-WT sequence was used as PNA probe.

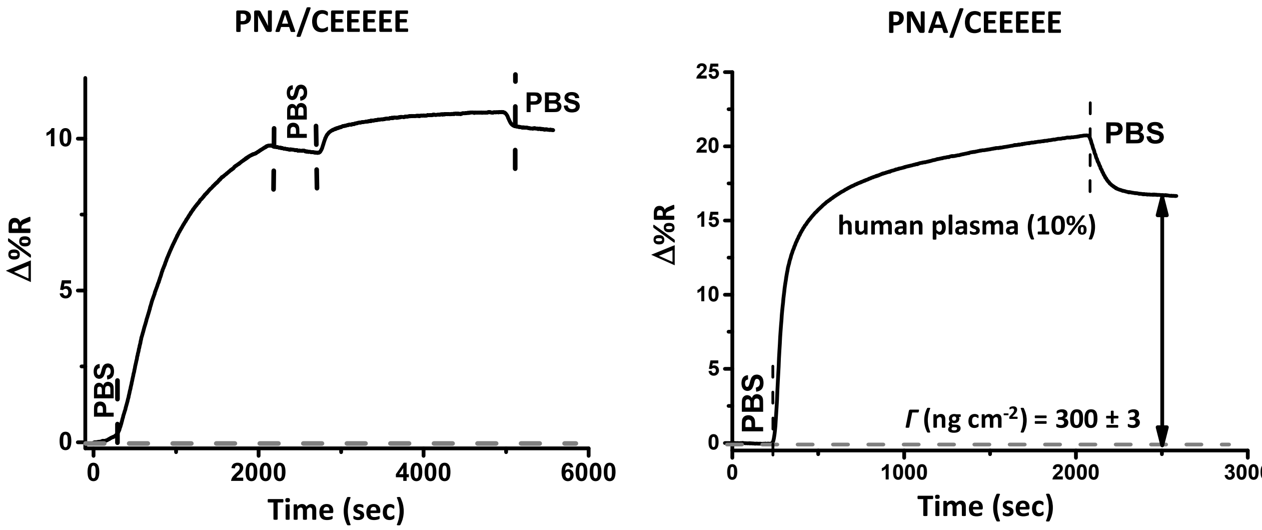

Figure S10. SPRI responses and kinetic profiles detected for the immobilization of PNA/CEEEEE on gold surface and SPRI adsorption curves of plasma proteins on PNA/CEEEEE after the exposure of diluted human plasma solution (10%) for 30 minutes. PNA-WT sequence was used as PNA probe. The antifouling tests were replicated in three independent experiments for each layer.

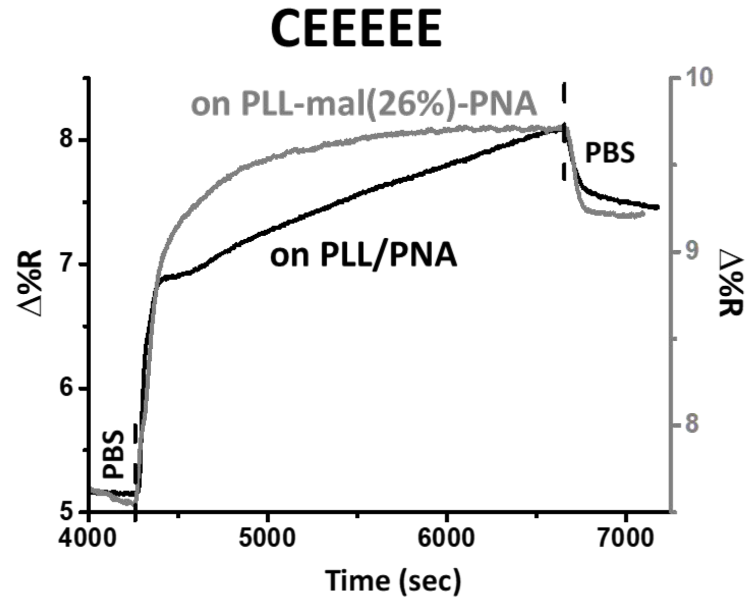

Figure S11. SPRI kinetic profiles detected for the immobilization of CEEEEE on PLL/PNA (black line) and PLL-mal(26%)-PNA (grey line) surfaces.

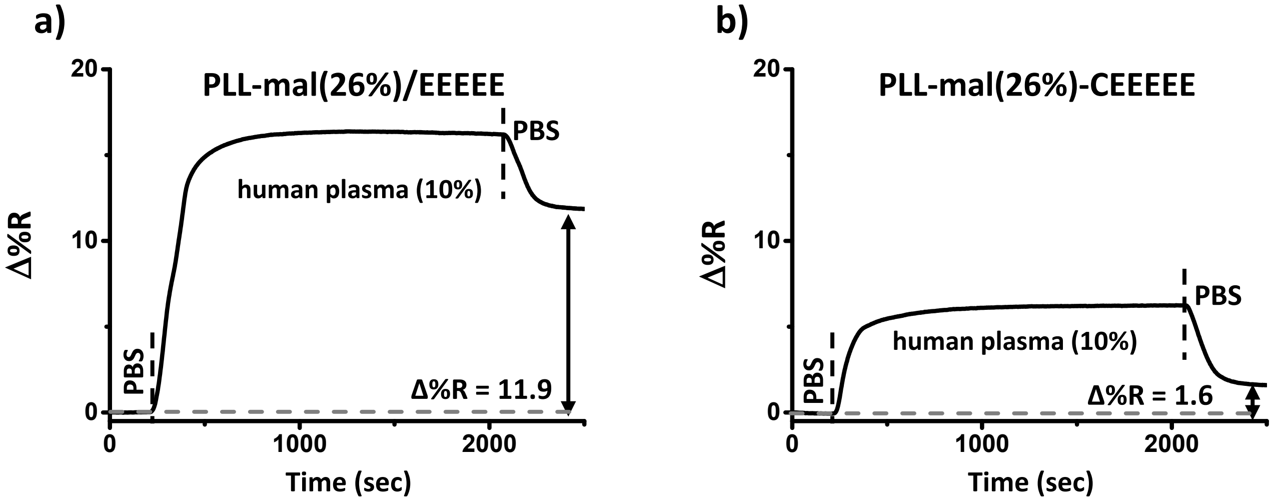

Figure S12. SPRI kinetic data for the adsorption of 10% human plasma on (a) PLL-mal(26%)/EEEEE and (b) PLL-mal(26%)-CEEEEE. PNA-WT sequence was used as PNA probe.

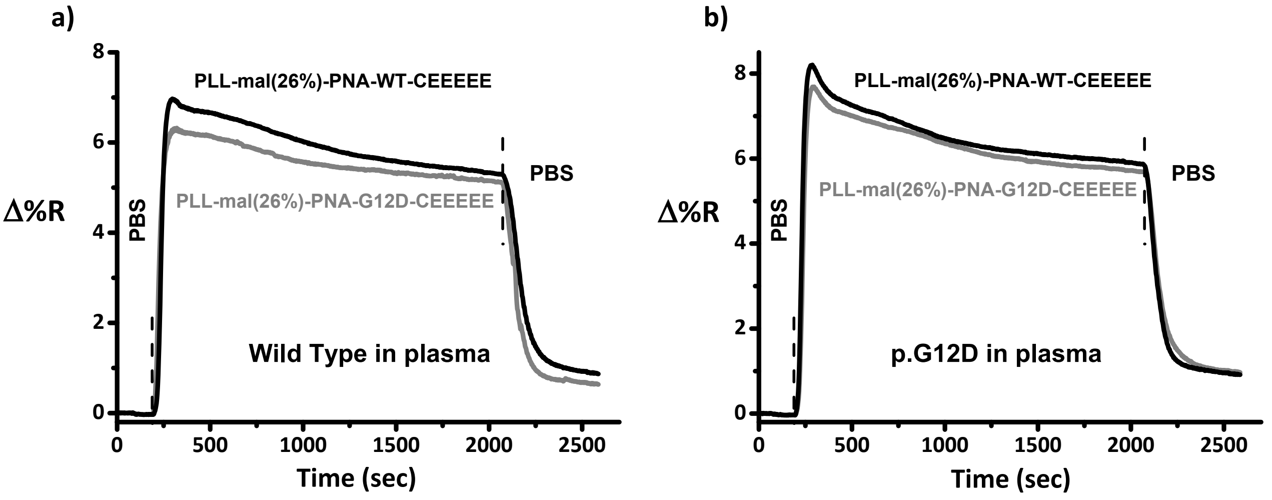

Figure S13. (a) Experimental SPRI curves, in percent reflectivity (Δ%R) over time, of wild-type gDNA on PLL-mal(26%)-PNA-CEEEEE layers bearing PNA-WT (black solid line) and PNA-G12D (grey solid line), respectively using 5 pg μL^{-1} of ctDNA fragments in diluted human plasma samples. (b) Experimental SPRI curves, in percent reflectivity (Δ%R) over time, of p.G12D gDNA on PLL-mal(26%)-PNA-CEEEEE layers bearing PNA-WT (black solid line) and PNA-G12D (grey solid line), respectively, using 5 pg μL^{-1} of ctDNA fragments in diluted human plasma samples.

Table S3. $\Delta\%R_{PNA\ G12D}$, $\Delta\%R_{PNA\ WT}$ and $\Delta\%R_{PNA\ G12D}/\Delta\%R_{PNA\ WT}$ values obtained from twelve replicated experiments aimed at detecting 5 pg μL^{-1} solutions of WT and G12D non-amplified genomic DNAs in human plasma samples after 1000 s of adsorption of conjugated AuNPs. The ratio considers SPRI responses (Δ%R) is referred to the PNA G12D probe ($\Delta\%R_{PNA\ G12D}$) and the PNA WT probe ($\Delta\%R_{PNA\ WT}$), respectively when the same DNA target was detected. The mean $\Delta\%R_{PNA\ G12D}/\Delta\%R_{PNA\ WT}$ ratio value is reported in Fig. 6.

Sample	Δ%R PNA G12D / Δ%R PNA WT	Sample	Δ%R PNA G12D / Δ%R PNA WT
	1.21		0.96
	1.26		0.91
	1.02		0.90
	1.04		0.93
	1.49		0.89
gDNA G12D	1.39	gDNA WT	0.70
	1.03		-
	1.08		0.86
	1.28		0.73
	1.21		-
